# Deficiency of B vitamins leads to cholesterol-independent atherogenic transformation of the aorta

**DOI:** 10.1016/j.biopha.2022.113640

**Published:** 2022-09-05

**Authors:** Gunter Almer, Peter Opriessnig, Heimo Wolinski, Gerhard Sommer, Clemens Diwoky, Margarete Lechleitner, Dagmar Kolb, Vladimir Bubalo, Markus S. Brunner, Andreas N. Schwarz, Gerd Leitinger, Gabriele Schoiswohl, Gunther Marsche, Tobias Niedrist, Silvia Schauer, Wolfgang Oswald, Andrea Groselj-Strele, Margret Paar, Gerhard Cvirn, Gerald Hoefler, Gerald N. Rechberger, Markus Herrmann, Sasa Frank, Gerhard A. Holzapfel, Dagmar Kratky, Harald Mangge, Gerd Hörl, Oksana Tehlivets

**Affiliations:** a Clinical Institute for Medical and Chemical Laboratory Diagnostics, Medical University of Graz, Graz, Austria; b Division of General Neurology, Department of Neurology, Medical University of Graz, Graz, Austria; c Division of Pediatric Radiology, Department of Radiology, Medical University of Graz, Graz, Austria; d Institute of Molecular Biosciences, University of Graz, Graz, Austria; e Institute of Biomechanics, Graz University of Technology, Graz, Austria; f Gottfried Schatz Research Center, Molecular Biology and Biochemistry, Medical University of Graz, Graz, Austria; g Gottfried Schatz Research Center, Cell Biology, Histology and Embryology, Medical University of Graz, Graz, Austria; h Center for Medical Research, Ultrastructure Analysis, Medical University of Graz, Graz, Austria; i Division of Biomedical Research, Medical University of Graz, Graz, Austria; j Department of Pharmacology and Toxicology, University of Graz, Graz, Austria; k Otto Loewi Research Center, Pharmacology, Medical University of Graz, Graz, Austria; l Diagnostic and Research Institute of Pathology, Medical University of Graz, Graz, Austria; m Department of Surgery, Clinical Division of Vascular Surgery, Medical University of Graz, Graz, Austria; n Center for Medical Research, Computational Bioanalytics, Medical University of Graz, Graz, Austria; o Otto Loewi Research Center, Division of Medicinal Chemistry, Medical University of Graz, Graz, Austria; p Department of Structural Engineering, Norwegian University of Science and Technology, Trondheim, Norway; q Division of General Radiology, Department of Radiology, Medical University of Graz, Graz, Austria

**Keywords:** Atherosclerosis, Rabbits, Balloon injury, B vitamins, Homocysteine

## Abstract

Atherosclerosis, the leading cause of cardiovascular disease responsible for the majority of deaths worldwide, cannot be sufficiently explained by established risk factors, including hypercholesterolemia. Elevated plasma homocysteine is an independent risk factor for atherosclerosis and is strongly linked to cardiovascular mortality. However, the role of homocysteine in atherosclerosis is still insufficiently understood. Previous research in this area has been also hampered by the lack of reproducible *in vivo* models of atherosclerosis that resemble the human situation. Here, we have developed and applied an automated system for vessel wall injury that leads to more homogenous damage and more pronounced atherosclerotic plaque development, even at low balloon pressure. Our automated system helped to glean vital details of cholesterol-independent changes in the aortic wall of balloon-injured rabbits. We show that deficiency of B vitamins, which are required for homocysteine degradation, leads to atherogenic transformation of the aorta resulting in accumulation of macrophages and lipids, impairment of its biomechanical properties and disorganization of aortic collagen/elastin in the absence of hypercholesterolemia. A combination of B vitamin deficiency and hypercholesterolemia leads to thickening of the aorta, decreased aortic water diffusion, increased LDL-cholesterol and impaired vascular reactivity compared to any single condition. Our findings suggest that deficiency of B vitamins leads to atherogenic transformation of the aorta even in the absence of hypercholesterolemia and aggravates atherosclerosis development in its presence.

## 1 Introduction

Cardiovascular disease, the leading cause of death worldwide, arises mostly due to the development of atherosclerosis [1]. However, only half of all atherosclerosis cases today can be explained by known risk factors such as hypercholesterolemia [1]. Rabbits and mice are the most common animal models used to study atherosclerosis [2]. Whereas mice develop atherosclerosis only after genetic manipulation, e.g. apolipoprotein E (ApoE) or low-density lipoprotein receptor (Ldlr) knockout mice [3], rabbits on a high-fat diet spontaneously develop foam cell-rich plaques (fatty streaks) resembling human atheroma [4]. Injury to the arterial wall shortens the time required for advanced lesions to form in response to hyperlipidaemia [4]. In most cases, vascular injury in atherosclerotic animal models is achieved by repeated retraction of an inflated balloon tip catheter within an artery [5]. Here, we developed an automated system for balloon-mediated injury (JURY) and compared this technique with the classical manual injury of the aorta in the rabbit atherosclerosis model. JURY controls both the balloon pressure and catheter retraction speed and can therefore standardize and optimize vessel wall injury. Consequently, our system can be used to investigate the interplay of various risk factors for atherosclerosis.

Hyperhomocysteinemia (HHcy), characterized by elevated homocysteine (Hcy) levels in the plasma, is an independent risk factor for atherosclerosis. HHcy increases the risk when combined with hypercholesterolemia, and is closely associated with cardiovascular mortality [6,7]. In accordance, induction of HHcy by dietary deprivation of folate, vitamins B_6_ and B_12_, high methionine intake, or genetic block in Hcy metabolization exacerbates atherosclerosis in mouse models [8,9]. Choline is also required for Hcy metabolization and its deficiency is linked to HHcy [10]. HHcy is present in 5–10% of the general population, in up to 30% of the elderly and in 70% of men over 80 years of age [6,11,12]. Two-thirds of HHcy incidence is due to deficiency of vitamins required for Hcy metabolization [13]. Here, we show that a diet free from vitamin B_12_ and reduced in folate (20%), vitamin B_6_ (20%), and choline (10%) (VCDD) required for Hcy metabolization leads to atherosclerosis aggravation in the presence of hypercholesterolemia and atherogenic transformation of the aorta in its absence in the balloon-injured rabbit model of atherosclerosis. In summary, this study demonstrates i) an innovative technique for vessel wall injury, ii) a new atherosclerotic rabbit model that can be used to study lipid- and Hcy-related risk factors simultaneously, and iii) a combination of both to study atherosclerosis.

## 2 Materials and methods

### 2.1 JURY device for pressure- and retraction-controlled balloon denudation in rabbits

The JURY device for pressure- and retraction-controlled vessel wall injury was developed for use with balloon tip catheters in rabbits. Various Fogarty embolectomy catheters (3F40, 3F80, 4F40 and 4F80; mpö pfm GesmbH, Austria) were tested and selected according to the average size of the adult rabbit aorta abdominalis. *In vitro* tests showed that the Fogarty 4F40 catheter enables the best mechanical pressure control and that a threshold pressure of 1.2 bar is required to expand the balloon of the 4F40 catheter.

### 2.2 Animal experiments

All animal experiments were approved by the Austrian Federal Ministry of Education, Science and Research (BMWF-66.010/01 1 I-II/3b/2012 and 66.010_0070-V_3b_2018). For the study, 4- to 6-month old male rabbits (New Zealand White rabbits (NZW rabbits), Charles River (France), Schitkowitz (Austria) and Hyla/Wildtype crossbreds (cbHyla, Schitkowitz Austria)) were used. Prior to the studies, the rabbits were acclimatized for 2–4 weeks. Rabbits were set on special diets after or before surgery. The diets used included an unpurified complete chow diet (standard diet, SD) for full nutritional conditions and purified special diets either enriched with 1% cholesterol (HCD), deficient in vitamins and choline required for Hcy metabolization (no vitamin B_12_, 20% folate (2 mg/kg), 20% vitamin B_6_ (6 mg/kg) and 10% choline 128 mg/kg) (VCDD)) or combined VCD/HCD. Special purified diets were acquired from Sniff, Germany. Average daily diet intake was determined by weighing the remaining food after two days once per week for each animal. Weight was measured once a week. Six or eight weeks later, rabbits were sacrificed and the aortas dissected. The same sections were used for myography, MRI and other analyses. All specimens from myography and MRI were used for histology.

### 2.3 Vessel wall injury

Injury of the adult rabbit aorta abdominalis was performed by three techniques: the automated JURY-controlled injury, classical “blind” injury without pressure monitoring and manual pressure-adjusted injury (a modification of classical “blind” injury with monitoring and manual adjustment of the pressure). A detailed description of the JURY device and the surgery is provided in supplements 1 and 2.

1. *Classical “blind” injury:* manual injury, during which the pressure is regulated manually by thumb press on a syringe; no display of pressure data for the operator; manual retraction at undefined speed. To compare pressure curve in classical “blind” injury with pressure curves in manual pressure-adjusted injury and JURY-controlled injury, the catheter during classical “blind” injury was connected to a 1 ml Luer-Lock syringe and the JURY device with a pressure sensor in between. Only during inflation of the balloon was the operator informed by the assistant when the target pressure was reached, otherwise the operator could only maintain the pressure according to their feeling (“blind”).
2. *Manual pressure-adjusted injury:* a modification of the classical “blind” injury with monitoring and manual adjustment of the pressure by the operator based on the displayed pressure curve; manual retraction at undefined speed. The catheter was connected to a 1 ml Luer-Lock syringe and the JURY device with a pressure sensor in between. The balloon pressure was displayed on a computer monitor in real-time.
3. *JURY-controlled injury:* fully automated JURY-controlled injury with pressure being displayed, recorded and adjusted automatically, JURY-controlled retraction at preset speed. For this technique, the catheter is locked to a pressure sensor (Pendotech, USA) on a 1 ml Luer-Lock syringe that is firmly clamped to the sled of the JURY device (Fig. S2). The desired target pressure and the retraction speed are entered into the graphical user interface of the JURY controller software (Arduino, USA). After reaching the target pressure, which takes a few seconds, retraction of the catheter is initiated. After reaching the desired end position, the balloon is deflated and the catheter is gently removed. Initiated by the operator, balloon inflation, retraction at constant pressure and constant speed as well as deflation are all performed automatically by the JURY device.

**Fig. 1:**
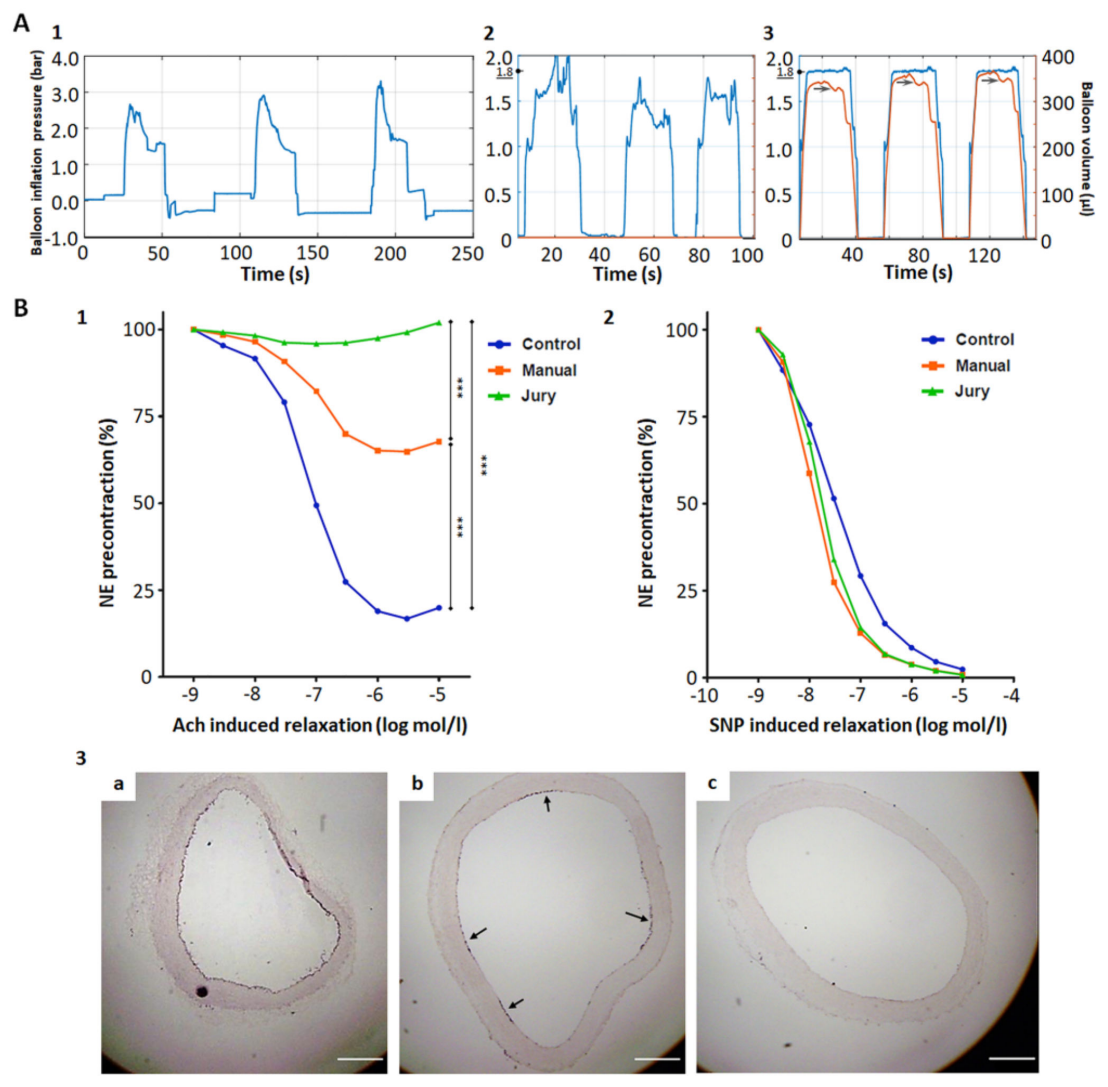
Development of the JURY-controlled balloon injury technique and comparison with existing vessel wall injury techniques. One rabbit per condition underwent vessel wall injury of the aorta abdominalis at a target pressure of 1.8 bar. Real-time pressure curves of three injury cycles (inflation-retraction-deflation) are shown. Black round arrows mark target pressure. Gray arrows mark reduction of balloon volume due to aorta narrowing. A1 Classical “blind” injury: no pressure monitoring and adjustment, manual retraction, pressure was recorded by JURY for comparison with other techniques. A2 Modification of the classical “blind” injury – manual pressure-adjusted injury: pressure was manually adjusted by the operator based on pressure curve monitoring, manual retraction. A3 Fully automated injury: JURY-controlled pre-adjusted pressure, JURY-controlled pre-adjusted retraction. Balloon volume is shown in μl. B Effects of JURY-controlled and manual pressure-adjusted injury on endothelium under mild injury conditions. Three rabbits per condition underwent surgical intervention. Vessel wall injuries were performed at minimal balloon inflation pressure of 1.2 bar with single retraction. The next day, rabbits were sacrificed and the abdominal aorta dissected at the area of denudation. Two untreated rabbits were used as control. Shown is the relaxation of freshly dissected aortic sections, repeated measurements (in total 6–8 per rabbit) with acetylcholine (Ach) (B1) and sodium nitroprusside (SNP) (B2) after precontraction with norepinephrine (NE). *p 0.05; **p 0.01; ***p 0.001. Immunohistological staining (B3) of endothelial cells (CD31) of untreated control aorta with intact endothelial layer (B3a), aorta injured by manual pressure-adjusted technique with partially denuded endothelial layer (B3b) (black arrows indicate intact endothelial areas) and aorta automatically injured using the JURY device with completely denuded endothelial layer (B3c). Images are representative for each group. Scalebars = 500 μm. Significant differences are shown in S7.

To compare the injury protocols described above 3 NZW rabbits were used. In the first animal, the operator performed the balloon injury “blind” without monitoring the pressure curve. In the second animal, the operator monitored the pressure curve during the denudation, and manually adjusted the pressure to 1.8 bar. The catheter was retracted manually (undefined speed) in the first and second animals. In the third animal, the JURY device was pre-adjusted to maintain a pressure of 1.8 bar and a constant retraction speed of 5 mm/s. In all cases, pressure and, if applicable, motor step data were recorded by the JURY device in real-time. The comparison of the methods is shown in Fig. 1. Due to high variation in the target pressure, the classical “blind” injury protocol was not further used.

Subsequently, abdominal aortas were injured in 6 NZW rabbits at a target pressure of 1.2 bar (minimal pressure for inflating the balloon) and a single retraction: 3 rabbits were treated according to the manual pressure-adjusted injury protocol and 3 rabbits according to the automated JURY-controlled injury protocol (constant retraction speed of 5 mm/s). Aortas from two untreated rabbits were used as controls. The following day, rabbits were sacrificed and the aorta abdominalis dissected in the area of the denudation to assess the injury.

In the next step, atherosclerotic plaque formation was examined in rabbit groups of 4–6 (NZW rabbits and cbHyla). Aortas were injured by manual pressure-adjusted or JURY-controlled injury techniques. The injury conditions used were either 1.2 bar (mild injury: minimal pressure; 1x pull-back) or 1.8 bar (severe injury: higher pressure; 3x pull-backs). After the injury, all rabbits were fed HCD for 6 weeks.

NZW rabbits were used to compare the effects of the modified diets on atherosclerotic transformation of the aorta. The rabbits were divided into 4 groups of 8 animals each. All vessel wall injuries were performed automatically with one retraction by the JURY device at 1.8 bar and a 60% reduced retraction speed of 2 mm/s to intensify single-applied injury. In the HCD, VCDD and VCD/HCD groups, the animals were preconditioned before the surgery (VCDD was initiated two weeks and HCD one week before vessel wall injury). One day after injury, the special diets were resumed and fed for another 8 weeks.

### 2.4 Plasma Hcy, total cholesterol and triglyceride levels

Methanol (MeOH), water (MS-grade) and acetonitrile were purchased from Merck, USA. 1,4-dithiothreitol (DTT), trifluoroacetic anhydride and ammonium acetate were purchased from Sigma, USA. Formic acid was purchased from Roth, Germany. L-Hcy from Sigma was used as standard. DL-Hcy-d4 from CDN isotopes, Canada was used as an internal standard. All standard stock solutions were prepared in H_2_O + 0.1% formic acid and stored at 20 °C until use. Blood was taken from the ear vein or by heart puncture after sacrifice, collected into EDTA or Lithium Heparin Vacuette® tubes (Greiner, Germany) and centrifuged for 15 min at 4 °C and 2000 g. Blood plasma was immediately aliquoted and frozen at 80 °C. To analyse the plasma Hcy levels, 100 μl of each plasma sample was mixed with 25 μl of 20 mmol/l DL-Hcy-d4 as internal standard and 10 μl of 0.5 mol/l DTT and incubated for 15 min at RT. Then, 300 μl of precipitation reagent (acetonitrile + 0.1% formic acid + 0.05% trifluoroacetic acid) was added, the samples were centrifuged for 5 min at 13,000 rpm at 4 °C and 125 μl of the supernatant was transferred into an autosampler vial. The solvent was then evaporated for about 30–45 min under a stream of nitrogen and the samples were reconstituted in 125 μl of H_2_O + 0.1% formic acid. For the absolute quantification of Hcy in the plasma, a dilution series for a calibration curve in the range from 1.4138 to 113.1034 μmol/l Hcy was prepared, followed by the preparation of quality controls (QC) at 94.25 μmol/l, 11.31 μmol/l and 1.885 μmol/l Hcy. Plasma Hcy levels were analyzed using a 1290 Infinity UHPLC coupled to a 6470 Triple-Quadrupole mass spectrometer (Agilent, USA) using a BEH C_18_ column (3.0 mm × 150 mm; 1.7 μm) (Waters, USA) with 50 °C column temperature, 5 μl injection volume and a constant flow rate of 200 μl/min under the control of Agilent MassHunter Workstation Data Acquisition software. H_2_O + 0.1% formic acid (A) and methanol + 0.1% formic acid (B) were used as solvents. 100% solvent A was held for 2 min, followed by a change to 100% solvent B over the next 2 min, which was held for an additional 3.5 min. Re-equilibration was carried out by changing to 100% solvent A within 3 s, followed by 3.5 min at 100% solvent A. Total running time was 11 min. Hcy and Hcy-d4 as internal standards were analyzed in MRM mode. The dwell time for all transitions was 60 ms, the fragmentor voltage 80 V and the cell acceleration voltage 4 V. The transitions *m/z* 136–56 for Hcy and *m/z* 140–94 for Hcy-d4, with a collision energy of 19 V and 15 V, respectively, were used as quantifiers. The transitions *m/z* 136–73 and 90 were the qualifiers for Hcy, *m/z* 140–77 and 59 for Hcy-d4 with a collision energy of 15 V and 19 V, respectively. Plasma total cholesterol and triglyceride levels were analysed using a cobas 8000 modular routine analyser (Roche, Austria).

### 2.5 Lipoprotein analysis

Plasma from rabbits of each dietary group was pooled and diluted with PBS if necessary. 200 μl of each plasma pool was subjected to fast protein liquid chromatography (FPLC) on a Pharmacia FPLC system (Pfizer Pharma, Germany) equipped with a Superose 6 (30x1cm) column (Amersham Biosciences, USA). The lipoproteins were eluted with 10 mM Tris-HCl, 1 mM EDTA, 0.9% NaCl and 0.02% NaN_3_ (pH 7.4) in fractions of 0.5 ml/min for 60 min. Total cholesterol and triglyceride concentrations were determined enzymatically. To enhance sensitivity, the reaction buffers were supplemented with sodium 3,5-dichloro-2-hydroxy-benzenesulfonate.

### 2.6 Biaxial extension tests

The biomechanical properties of the aortas were determined by biaxial strain tests of square-shaped aortic specimens with a side length of approx. 8 mm, the sides being aligned in the circumferential and axial directions of the aorta. The specimens were mounted to the carriages of the biaxial extension stage and immersed in a phosphate-buffered physiological solution (PBS) at 37 °C. During testing, the specimens were subjected to various loading protocols in order to determine the physiological deformations of the tissue. Different stretches (1.1–1.6 in 0.1 increments) were applied consecutively, starting with the lowest stretch until the tissue failed. For each stretch level, four preconditioning cycles and one measuring cycle were performed with different loading ratios (1(circ.):1(axial), 1:0.75; 1:0.5; 0.75:1; 0.5:1) with an actuator speed of 3 mm/min. Each measuring cycle consisted of a loading and an unloading path. It is crucial to use different load ratios between the circumferential and axial directions in order to cover the physiological deformations and to capture the direction-dependent material response of the aortas by means of biaxial extension tests. In addition, this so-called ‘true’ biaxial test approach results in a unique set of constitutive parameters for the specimen to be tested. The forces of load cells, positions of carriages and distances between special tissue markers (which act as gage markers) recorded by a videoextensometer were recorded continuously. Cauchy stress versus stretch ratio plots were generated from these data sets. The results were found to be very sensitive to the initial preloads. In order to obtain reproducible results, a prestress of about 0.1 kPa in terms of engineering stress (corresponds to 5 mN) was applied to each specimen.

### 2.7 Myography

Aortic rings were prepared from the abdominal aorta. The aortic segments were rapidly cleaned from the surrounding tissue, cut into 2 mm rings and transferred to the physiological buffer described below. The rings were mounted on pins connected to a micrometer and a force transducer. The experiments were carried out in myograph chambers (620 M Multi Wire Myograph System; Danish MyoTechnology, Denmark) filled with a modified Krebs-Ringer bicarbonate buffer solution (KRS) consisting of 119 mM NaCl, 4.7 mM KCl, 1.2 mM KH_2_PO_4_, 1.2 mM MgCl_2_, 2.5 mM CaCl_2_, 25 mM NaHCO_3_, 0.03 mM EDTA-Na_2_ and 5.5 mM D-glucose. The rings were kept in an open bath at 37 °C, while the KRS was continuously oxygenated with 95% O_2_ and 5% CO_2_ to keep the pH at 7.4. The maximal active tension was achieved by contracting the rings with the modified KRS with high K^+^ concentration (65 mM NaCl, 59 mM KCl, 1.2 mM MgSO_4_, 1.2 mM KH_2_PO_4_, 2.5 mM CaCl_2_, 25 mM NaHCO_3_, 0.03 mM EDTA-Na_2_ and 5.5 mM D-glucose). After reaching maximal isometric tension, the K^+^ was washed out and the rings were allowed to relax in a physiological buffer. The rings were then gradually contracted with increasing concentrations of norepinephrine (1 nM – 0.3 μM) to reach 80% of the maximum norepinephrine-induced contraction and then relaxed with increasing concentrations of acetylcholine chloride (1 nM – 0.3 μM) to assess endothelium-dependent relaxation. In order to examine endothelium-independent relaxation, the rings were washed and constricted with norepinephrine to 80% of the maximal constriction, and subsequently exposed to increasing concentrations of the nitric oxide donor, sodium nitroprusside (0.1–30 nM). Relaxation values were expressed as a percentage of the norepinephrine-induced contraction.

### 2.8 MRI analysis

**Fig. 2:**
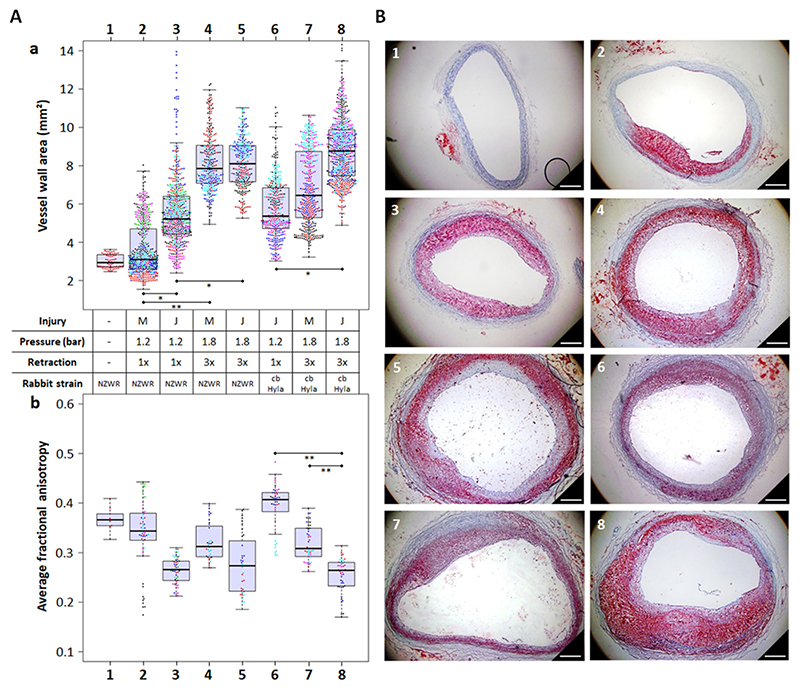
Atherosclerotic plaque development in two rabbit strains injured by JURY-controlled (J) vs manual pressure-adjusted technique (M). Groups of 4–6 animals (NZW (column 1–5) and cbHyla rabbits (column 6–8)) were injured either once at minimum pressure of 1.2 bar (A conditions 2, 3 and 6) or three times at 1.8 bar (A conditions 4, 5, 7 and 8) either manually (M) or with the JURY device (J) at a retraction speed of 5 mm/s. A single untreated NZW rabbit (A column 1) served as a reference and was not used in statistical evaluations. After 6 weeks of HCD, animals were sacrificed and aortas dissected. A MRI analysis of vessel wall area (AI) and fractional anisotropy (AII) of aortas from rabbits injured by JURY-controlled vs manual pressure-adjusted technique. Individual measuring points for each rabbit per group can be distinguished by the respective colors. Bold lines of boxes represent medians. *p 0.05; **p 0.01; ***p 0.001. B Comparative histology of aortic specimens (image index numbers correspond to conditions displayed in A). Identical aortic sections were cut into 8 smaller rings for cryo-sectioning and stained with oil red O (red) for neutral lipids and hematoxylin (blue) for cell nuclei. Images are representative for each group. Scalebars= 500 μm. Significant differences are shown in S7.

**Fig. 3:**
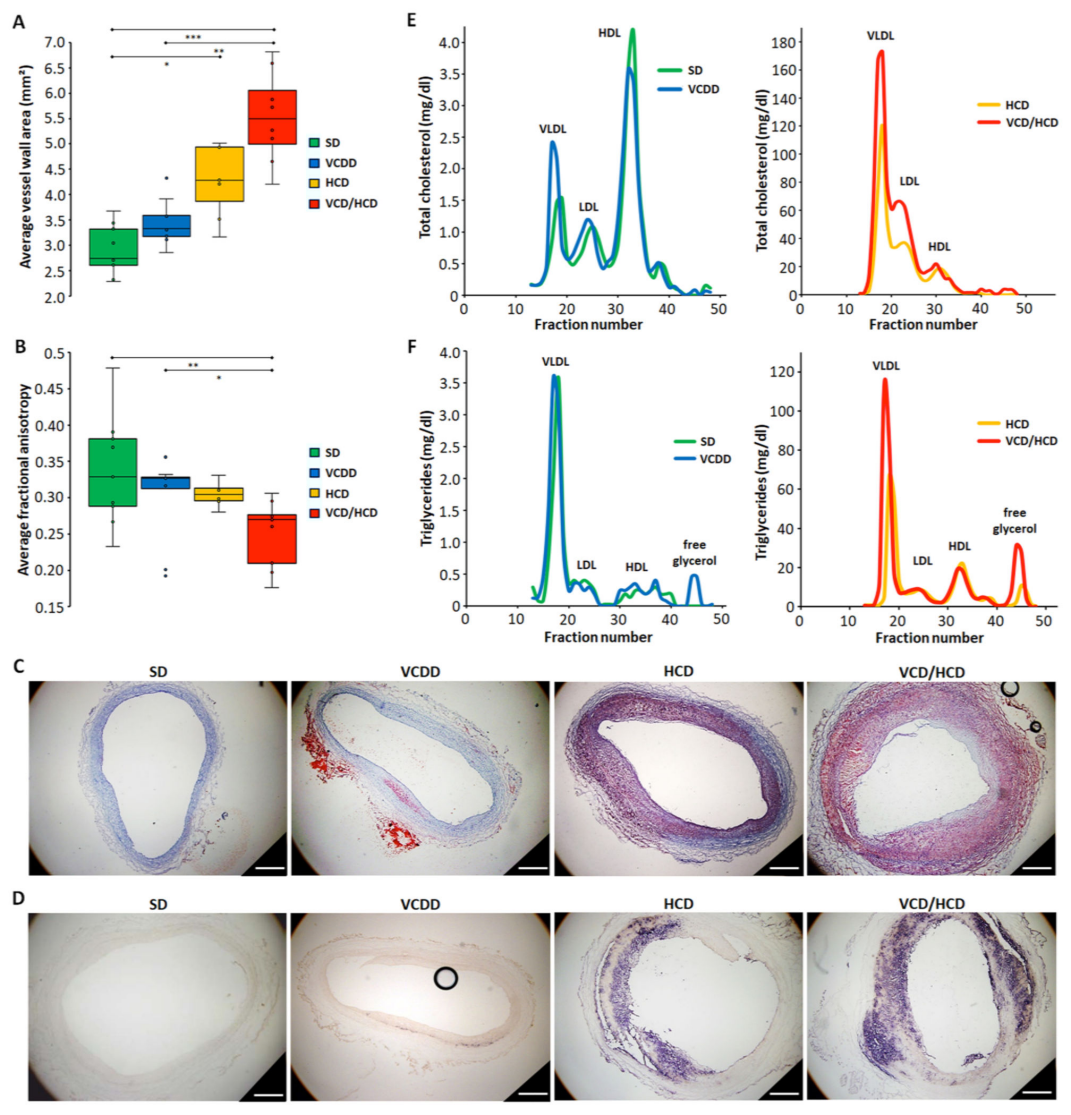
Atherosclerotic plaque development in JURY-injured aortas from rabbits fed different diets. Groups of 8 NZW rabbits were fed different diets and subjected to automated JURY-controlled injury at a pressure of 1.8 bar, single retraction and a 60% reduced retraction speed of 2 mm/s to intensify single-applied injury. VCDD was initiated two weeks and HCD one week before vessel wall injury for preconditioning. After 8 weeks post injury, animals were sacrificed and aortas dissected. A-B MRI analysis of average vessel wall area (A) and fractional anisotropy (B). C-D Representative histological sections stained with oil red O (red) for neutral lipids and hematoxylin (blue) for cell nuclei (C), and anti-rabbit RAM11 antibody (purple) for macrophages and neutral red (background) (D). E-F Lipoprotein profile of total cholesterol (E) and triglycerides (F) after fast protein liquid chromatography separation of pooled plasma samples, (n = 3–4). *p 0.05; **p 0.01; ***p 0.001. Scalebars = 500 μm. Significant differences are shown in S7.

Evaluation of plaque area: A 5 cm piece of dissected aorta was washed with PBS (pH 7.4) and immersed in perfluoropolyether (Fomblin®, Solvay, Italy) in a 1 ml Luer-Lock syringe without air bubbles. Fomblin®, a contrast agent without MR signal, was added to minimize magnetic susceptibility effects at air-tissue boundaries [14]. The specimens were scanned with a 15.2 Tesla small animal MRI (Biospec 152/11, Bruker, Germany). For segmentation of the aortic sections, a proton density-weighted, fat-saturated 2D fast spin echo sequence with the following parameters was used: TR/TE = 1500/2.9 ms, turbo factor = 1, slice thickness = 500 μm, in-plane resolution = 100 μm x 100 μm, imaging matrix = 200 × 200, the number of segments was between 59 and 120 slices (Fig. 2). A slightly different setting was used to assess plaque area in rabbits fed different diets (Fig. 3). To assess the plaque area in rabbits fed different diets, aortic sections of 1 cm in length were taken from approximately the same position from each animal and immersed in Fomblin®. A 7 Tesla small-animal scanner (Biospec 70/20, Bruker, Germany) equipped with a cryogenic cooled transceiver was used. The proton density-weighted, fat-saturated 2D fast spin echo sequence had the following parameters: TR/TE = 1900/6.64 ms, turbo factor = 2, slice thickness = 500 μm, in-plane resolution = 60 μm x 60 μm, imaging matrix = 200 × 200, 40 slices per aorta. For both studies, the mean cross-sectional area of the vessel wall across the entire specimens was analyzed by semi-automated segmentation with a custom built tool created in Matlab (MathWorks Inc, USA).

Diffusion tensor MRI: Within the same imaging session, a fat-saturated segmented diffusion tensor imaging protocol was used with the key parameters: EPI readout with 4 segments, TR/TE = 2000/21.13 ms, slice thickness = 800 μm, in-plane resolution = 80 μm x 80 μm, imaging matrix = 128 × 128, 9 slices. Water diffusion was analyzed in 120 isotropically distributed directions at a b-value of 1000 s/mm^2^. The average of 10 non-diffusion-weighted scans was used as a reference for calibrating the diffusion tensor. Diffusion tensor images including fractional anisotropy maps of the aortic cross-sections were calculated with the software supplied with the MRI (Paravision 6.1, Bruker, Germany). After the scans, the syringes were emptied and refilled with PBS containing 4% paraformaldehyde (PFA) for preservation and later histological analysis.

### 2.9 Histology

For cryosectioning, the aortic rings were washed in PBS (pH 7.4), after MRI or myography, embedded in Tissue Tek O.C.T T COMPOUND (VWR International, Austria) and frozen at 20 °C in a cryotom (Microm, Germany). Slices of 8 μm thickness were cut, transferred to SuperFrost® Plus glass slides (Thermo Scientific, Germany) and dried overnight.

For CD31 endothelial and RAM11 macrophage immunostaining, procedures were performed at RT using a Polink-2 Plus Mouse Detection System kit (GBI Labs, USA). The slides were rinsed twice with PBS (5 min each time) and blocked with sterile-filtered PBS + 5% BSA for 30 min. The slides were then incubated with a mouse anti-human CD31 antibody (clone JC/70 A, Abcam, UK) at 1:20 dilution or an anti-rabbit RAM11 antibody (Dako/Agilent, USA) 1:50 dilution in PBS + 0.5% BSA, according to the Polink staining protocol. As substrate BCIP/NBT solution (GBI Labs, USA) was used, and the slides were counterstained with filtered neutral red solution (1% acetic acetate in dH_2_O, 10 mg/ml neutral red dye (Fluka Analytical, Switzerland) for 5 min. Finally, the slides were dried, and covered with Aquatex (Merck, USA) and a cover slip.

Oil red O neutral lipid staining was performed at RT. The slides were each rinsed with PBS, distilled water and 2-propanol solution (60% + 40% dH_2_O) for 5 min. The slides were then stained in freshly prepared and filtered oil red O solution (60% 2-propanol, 40% dH_2_O, 10 mg/ml oil red O dye, Sigma-Aldrich, USA) for 15 min, briefly de-stained with the same 2-propanol solution and washed in dH_2_O. Finally, the slides were counterstained for nuclei with Haemalaun nach Mayer (Gatt-Koller, Austria) for 2 min, washed with dH_2_O, dried, and covered with Aquadex (Merck, USA) and a cover slip. After drying, the slides were visualized in transmitted light mode of an Olympus BX51 Basic Fluorescence Microscope (Germany) with a DP71 camera. All images were taken with an UPlan Apo Infinity Corrected 4xobjective. Liver tissue was fixed in PBS pH 7.4 + 4% PFA and embedded in paraffin blocks. Sections were cut 3 μm thick using a microtome (Histocom, Micron HM440E, Switzerland), and then stained with hematoxylin and eosin according to standard protocols.

### 2.10 Sample preparation and multi-photon microscopy

Ring-like pieces of rabbit aorta were cut longitudinally under the stereomicroscope using razor blades and subsequently mounted between two coverslips so that the aortic intima was flat to the objective. Imaging was performed at the IMB-Graz Optical Imaging Resource using a picosecond laser (picoEmerald; APE, Germany) integrated in a Leica SP5 confocal microscope (Leica Microsystems, Germany). The laser delivers temporally and spatially overlapping pulse trains: a stable 1064 nm line, a tunable signal beam and an idler beam. For coherent anti-Stokes Raman scattering (CARS) imaging of densely packed neutral lipids in lipid droplets, the signal beam was tuned to 816.4 nm (2840 cm^-1^). The sample was illuminated simultaneously with the 1064 nm line, which led to the CARS signal of symmetrical CH_2_ stretching vibrations of esterified fatty acids in lipid droplets. For second harmonic generation (SHG) imaging of collagen, the signal beam was tuned to 880 nm. A two-channel, non-descanned detector (NDD) in epimode was used to detect CARS and SHG signals simultaneously. An SP 680 barrier filter (excitation light filter), an RSP 495 beamsplitter for two-channel separation of emitted light towards a 465/120 (‘SHG channel’) and a 650/210 (‘CARS channel’) bandpass filter was used. The wide range of the bandpass filters allows the additional detection of two-photon excited autofluorescence (TPAF) mainly of elastin in the ‘CARS channel’ and of elastin and cellular structures in the ‘SHG channel.’ The SHG signal from collagen and the CARS signal from lipid droplets were typically significantly stronger than the autofluorescence signal from elastin and cellular structures under the same imaging conditions. Optical sections were acquired using HCX IRAPO L 25xNA 0.95 water immersion and a sampling interval of 125 nm x 125 nm. Eight- to sixteen-times line averaging was applied to reduce image noise.

### 2.11 Electron microscopy

Aortic tissue was fixed in 2.5% (w/v) glutaraldehyde and 2% PFA (w/v) in 0.1 M cacodylate buffer, pH 7.4, for 2 h, and then post-fixed in 2% (w/v) osmium tetroxide for 2 h at room temperature (RT). After dehydration (in graded series of ethanol), tissues were infiltrated (ethanol and TAAB Embedding Resin, pure TAAB Embedding Resin) and placed in TAAB Embedding Resin (8 h), transferred into embedding molds, and polymerized (48 h, 60 °C). Ultrathin sections (70 nm) were cut with a UC 7 Ultramicrotome (Leica Microsystems, Austria) and stained with lead citrate for 5 min and platinum blue for 15 min. Electron micrographs were taken using a Tecnai G2 transmission electron microscope (FEI, Netherlands) with a Gatan Ultrascan 1000 charge coupled device (CCD) camera (20 °C, acquisition software Digital Micrograph, Gatan, Germany and Serial EM). Acceleration voltage was 120 kV.

### 2.12 Statistical analysis

Data are presented as line graphs or as box plots partially with single data points superimposed (e.g. vessel wall area), respectively. Sample sizes in figure legends refer to biological replicates (independent animals). Comparisons between two groups were done by Mann-Whitney U-test, if data violating normality distribution. In case of multiple group comparisons, analysis of variance (ANOVA) or Kruskal-Wallis test followed by Bonferroni post-hoc tests were applied. In the case of serial measurements (concentration), Greenhouse-Geisser-corrected two-way repeated-measures ANOVA was used. Generally, significant factorial designs were followed by pairwise comparisons that were corrected in case of multiple comparisons by Bonferroni post-hoc analyses. Data residual distribution was confirmed by Shapiro-Wilk’s test and visual data inspection by use of Q-Q plots, whereas homogeneity of variance was verified by Levene’s test. All reported P values are two-sided, and an α level of 0.05 was used throughout. Analyses were performed with SPSS 27.0 (SPSS Inc., Chicago, USA) or GraphPad Prism 8 (GraphPad Software LLC, Massachusetts, USA). The statistical analysis is shown in S7.

## 3 Results

### 3.1 Development of JURY-controlled balloon injury technique to improve animal models of atherosclerosis

Currently used techniques for inducing atherogenesis do not ensure controlled, reproducible denudation of the endothelium of the aorta and/or arteries. Here, we have developed and tested an automated device that enables reproducible injury by controlling pressure and retraction speed and, thereby, injury severity. The JURY system displays, records and controls the pressure of the inflated balloon in real-time and retracts the balloon at a predefined speed. In brief, a saline-filled 4F40 Fogarty catheter was inserted into the femoral artery and advanced into the aorta abdominalis. After reaching the predefined start position, the balloon was inflated and retracted with pre-adjusted speed over a distance of 9 cm. A detailed description of the device, surgery and the JURY-controlled injury is given in supplements 1 and 2. To optimize and diversify the injury procedure, two different target pressures, 1.2 and 1.8 bar, as well as single or repeated retractions of the catheter at constant speed were applied.

Initially, we compared three injury protocols: 1) classical “blind” injury, during which pressure and retraction speed were manually regulated without pressure monitoring, 2) modified classical “blind” injury, whereby the pressure is manually adjusted based on displayed pressure data, and 3) our fully automated pressure- and retraction-controlled injury system (JURY device) with pressure data and motor steps for balloon inflation/deflation being displayed, recorded and adjusted automatically. The JURY-controlled injury exhibited the lowest fluctuation in maintaining the target pressure (Fig. 1A). The classical injury without pressure monitoring ("blind") showed the highest, while the modified “blind” system showed medium pressure fluctuations. The JURY device achieved the lowest fluctuation because it compensates for anatomy-related variations in vessel diameter by decreasing the balloon volume during retraction, as observed at sites where the aorta abdominalis narrows (Fig. 1A3). Thus, vessel wall injury with the JURY device can be performed on vessels of different thickness (e.g. aortas from animals of different size and age) with identical mechanical stress. Moreover, we observed large variations in retraction speed with the manual techniques. A constant retraction speed was only achieved by the JURY system.

Comparison of the automated JURY-controlled and the manual pressure-adjusted injury (with pressure monitoring, manual pressure adjustment and manual retraction) revealed that the JURY device enables complete endothelial denudation already at 1.2 bar with single retraction, whereas the manual pressure-adjusted injury with the same settings resulted only in partial denudation of the endothelium (Fig. 1B3). Both injury types significantly reduced acetylcholine-induced relaxation of the aortic rings compared to untreated controls (Fig. 1B1, 2 way ANOVA, JURY, 95% CI [ 49.93, 35.85], p 0.001; manual, 95% CI [ 35.21, 17.54], p 0.001; injury type F(2,48)= 66.44, p 0.001). Importantly, the automated JURY-controlled injury also significantly reduced the acetylcholine-induced relaxation compared to the manual-pressure-adjusted injury (Fig. 1B1, JURY-manual, 95% CI [ 22.02, 11.01], p 0.001), possibly due to incomplete denudation, indicating that automated injury at low pressure and single retraction was more efficient than manual injury. Neither injury protocol altered sodium nitroprusside-induced relaxation compared to untreated controls (Fig. 1B2, JURY, 95% CI [ 4.865, 16.12], p = 0.593; manual, 95% CI [ 2.596, 18.16], p = 0.216) indicating that the damage was confined to the endothelium and did not affect the underlying smooth muscle cells. The statistical analysis is shown in S7. Immunohistological staining confirmed that JURY-controlled balloon injury resulted in complete denudation of the endothelial layer (Fig. 1B3c), whereas the manual pressure-adjusted injury with the same settings resulted only in partial denudation of the endothelium (Fig. 1B3b).

Analysis of atherosclerotic plaque development in rabbits fed a high cholesterol Western-type diet (HCD) for 6 weeks post-injury revealed that the automated JURY-controlled injury at 1.2 bar and single retraction led to significantly thicker and more homogenous atherosclerotic plaques than the manual pressure-adjusted injury with the same settings (Fig. 2A1 and B; condition 3 vs 2; U=32, p = 0.026, p_adj_=0.052). Increasing the balloon pressure to 1.8 bar and the number of retractions to three (severe injury protocol) significantly increased the thickness of the aortic wall in both JURY and manual pressure-adjusted injury (Fig. 2A1 and B; condition 5 vs 3; U=22, p = 0.038, p_adj_=0.076; condition 4 vs 2; U=24, p = 0.010, p_adj_=0.020, respectively) compared to minimal injury protocol (1.2 bar and single retraction). Under severe injury conditions, no differences in plaque size were observed between automated and manual injury in NZW rabbits (Fig. 2A1 and B; condition 5 vs 4, U=8, p = 1.000, p_adj_=2.000). JURY-controlled endothelial denudation led to comparable atherosclerotic plaque formation also in Hyla/Wildtype crossbreds (cbHyla) (Fig. 2A1 and B). The severe injury protocol significantly increased the thickness of the aortic wall compared to the minimal injury protocol when the JURY device was used in cbHyla rabbits (Fig. 2A1; condition 8 vs 6; U=24, p = 0.016, p_adj_=0.038). Notably, in cbHyla animals, JURY-controlled injury at 1.8 bar with three pull-backs resulted in more homogenous atherosclerotic plaque formation with non-significantly increased plaque thickness compared to the manual pressure-adjusted injury (Fig. 2A1 and B; condition 8 vs 7, U=20, p = 0.151, p_adj_=0.302).

Diffusion tensor magnetic resonance imaging (MRI) allows microscopic fiber orientation and cellular structure to be assessed by measuring the fractional anisotropy (FA) of water diffusion [15]. A higher FA corresponds to healthy intima/media tissue, whereas a low FA is associated with ageing and alteration of the arterial tissue microstructure [16,17]. In NZW rabbits, the JURY-controlled minimal or severe injury protocols resulted in non-significantly decreased FA compared to manual procedures with the same settings (Fig. 2A2; condition 3 vs 2, U=6, p = 0.065, p_adj_=0.130 and 5 vs 4; U=3, p = 0.200, p_adj_=0.400, respectively). In contrast, in cbHyla rabbits, both, the severe JURY-controlled protocol compared to the minimal JURY-controlled protocol, as well as the severe JURY-controlled protocol compared to severe manual protocol, led to significantly decreased FA (Fig. 2A2; condition 8 vs 6 and condition 8 vs 7; both U=0, p = 0.008, p_adj_=0.016).

### 3.2 B vitamin deficiency leads to atherogenic transformation of the aorta in the absence of hypercholesterolemia and aggravated atherosclerosis in its presence

Feeding JURY-injured rabbits a diet free from vitamin B_12_ and reduced in folate, vitamin B_6_, and choline (VCDD) in the absence or presence of hypercholesterolemia resulted in non-significant thickening of the aortic wall compared to chow diet or HCD, respectively (Fig. 3A, Kruskal-Wallis-Test, H=21.742, SD-VCDD, p = 0.157, p_adj_=0.944 and HCD-VCD/HCD, p = 0.143, p_adj_=0.855). However, more pronounced and highly significant aortic wall thickening in response to VCD/HCD in comparison to chow diet (p = 0.000, p_adj_=0.000) than in response to HCD alone (p = 0.011, p_adj_=0.068), suggests that VCDD indeed plays an important role in aortic thickening in the presence of HCD (Fig. 3A). Furthermore, VCD/HCD but not HCD alone leads to significantly lower FA than does chow diet (Fig. 3B, H=9.063, p = 0.006, p_adj_=0.035 and p = 0.563, p_adj_=1.000, respectively), further suggesting that VCDD is synergistic with HCD in atherogenic transformation of the aorta. VCDD alone led to cholesterol-independent macrophage and lipid droplets accumulation in the aortic neointima, and their abundance increased in response to VCD/HCD (Fig. 3C and D). Also, the low-density lipoprotein (LDL)-cholesterol increased in response to combined diet (Fig. 3E). These observations indicate that VCDD induces atherogenic alterations in the aorta - even in the absence of hypercholesterolemia - and increases atherogenicity of HCD. Very low-density lipoprotein (VLDL)-triglyceride and free glycerol were also elevated in response to VCD/HCD compared to HCD alone, indicating a synergistic effect of the combined diet not only on cholesterol but also on triglyceride metabolism (Fig. 3F). Notably, VCDD resulted in only a slight, non-significant accumulation of circulating Hcy, had no effect on total plasma cholesterol or triglyceride concentrations, food intake or weight gain, and did not result in any pathological changes of the liver compared to rabbits fed chow diet (supplement 3–5).

**Fig. 4:**
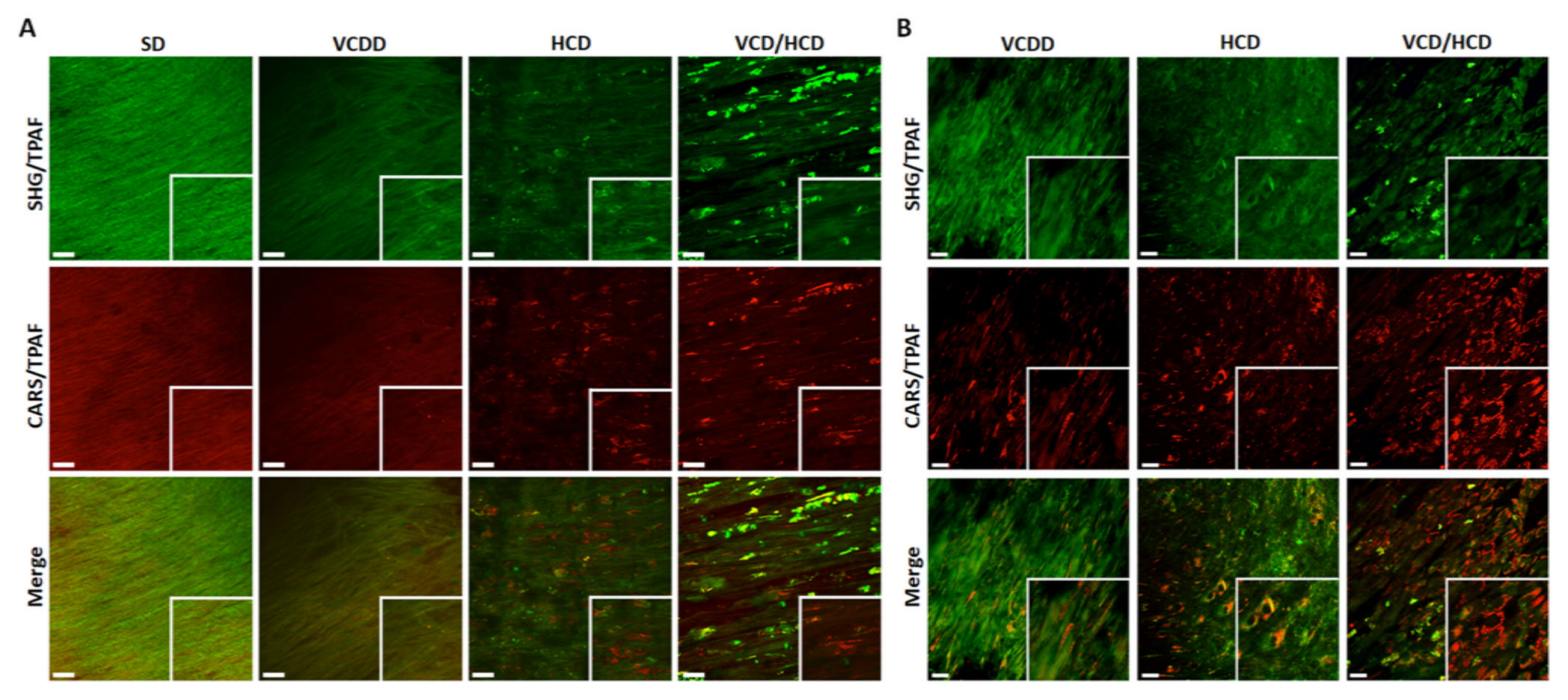
Second harmonic generation (SHG) and anti-Stokes Raman scattering (CARS) microscopy of aortic sections from rabbits fed different diets. A Collagen/elastin organization and lipid droplet accumulation. Representative images of collagen/elastin fiber organization (SHG/TPA) in top panel and lipid accumulation (CARS/TPAF) in middle panel in the aortic intima are shown. Strong signal occurring both in SHG/TPAF and CARS/TPAF channel is most likely derived from autofluorescence of dead/compromised cells (VCD/HCD condition). Penetration depth of non-linear optical imaging into non-cleared aortic samples is limited to ~150 μm. Presented images show structures in ~50–150 μm sample depth. B Accumulation of putative macrophages and neutral lipids at the surface of the intima, representative images. Here, fibrillar collagen/elastin structures were not detected in SHG/TPFA channel but putative autofluorescence of cell bodies. Strong CARS signals originating from spherical structures represent lipid droplets (CARS/TPAF). Scalebars = 30 μm.

**Fig. 5:**
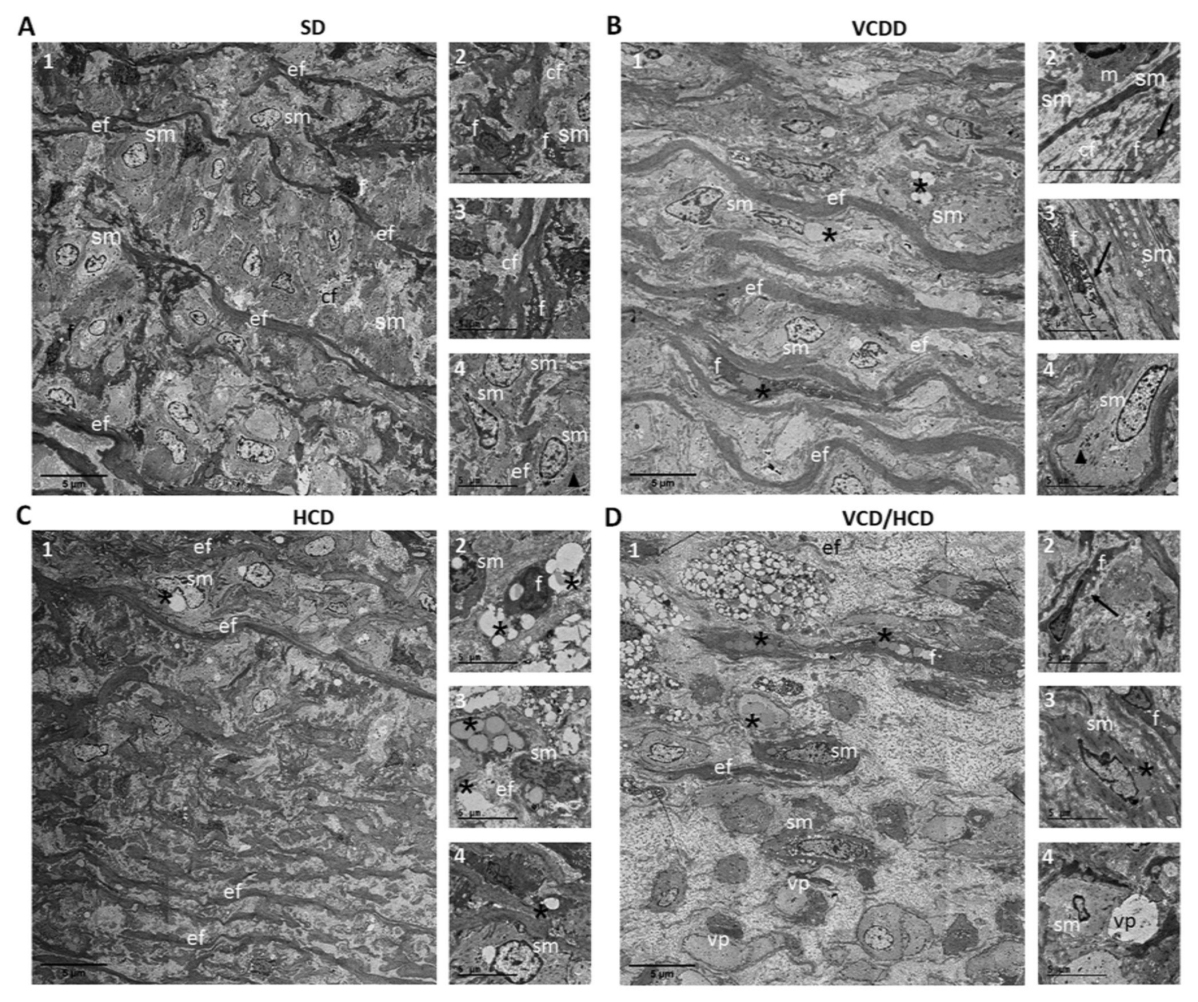
Representative transmission electron micrographs from aortic media of rabbits fed different diets. A1–4 SD. Elastic fibers (ef) appear continuous, smooth muscle cells (sm) are in close proximity, fibroblasts (f) and surrounding collagen fibers (cf) appear normal. Smooth muscle cells are recognized by their dense bodies (arrowhead in A4). B1–4 VCDD. Elastic fibers (ef) appear continuous, smooth muscle cells (sm) contain lipid droplets (asterisk). Fibroblasts (f) also indicate high amount of lipid droplets (asterisk) and dilated endoplasmic reticulum (arrows in 2 and 3). C1–4 HCD. Elastic fibers (ef) are continuous, both smooth muscle cells (sm) and fibroblasts (f) contain lipid droplets (asterisks) and are surrounded by collagen fibers (cf). D1–4 VCD/HCD. The spacing between elastic fibers (ef) is widened. Smooth muscle cells (sm) and fibroblasts (f) show accumulated lipid droplets (asterisks) and dilated endoplasmic reticulum (arrow in 2) respectively. Some putative smooth muscle fiber structures are swollen and void of compartments (void profiles, vp). Scalebars = 5 μm.

Second harmonic generation (SHG) and coherent anti-Stokes Raman scattering (CARS) microscopy revealed collagen/elastin disorganization, confirmed accumulation of lipids and showed accumulation of cellular structures reminiscent of macrophages in the aortic wall of JURY-injured rabbits fed VCDD (Fig. 4A and B). VCD/HCD resulted in further collagen/elastin disorganization as well as increased numbers of macrophages/foam cells in the plaque area compared to all other diets (Fig. 4A and B). Electron microscopic analysis of the aortic media further supported an independent impact of VCDD. Even in the absence of HCD, VCDD resulted in accumulation of lipid droplets and dilation of the endoplasmic reticulum (ER) in smooth muscle cells and fibroblasts in the aorta of JURY-injured rabbits. These changes were not observed in the rabbits fed chow diet (Fig. 5A and B). Dilated ER was only observed in rabbits fed VCDD, both in the absence and presence of HCD (Fig. 5B and D). Finally, in addition to lipid droplet and macrophage accumulation, VCD/HCD also led to wider spacing between elastic fibers and smooth muscle cells in the aortic media. These changes were not observed in chow diet-fed rabbits, thereby confirming the detrimental effects of VCD/HCD (Fig. 5D).

**Fig. 6:**
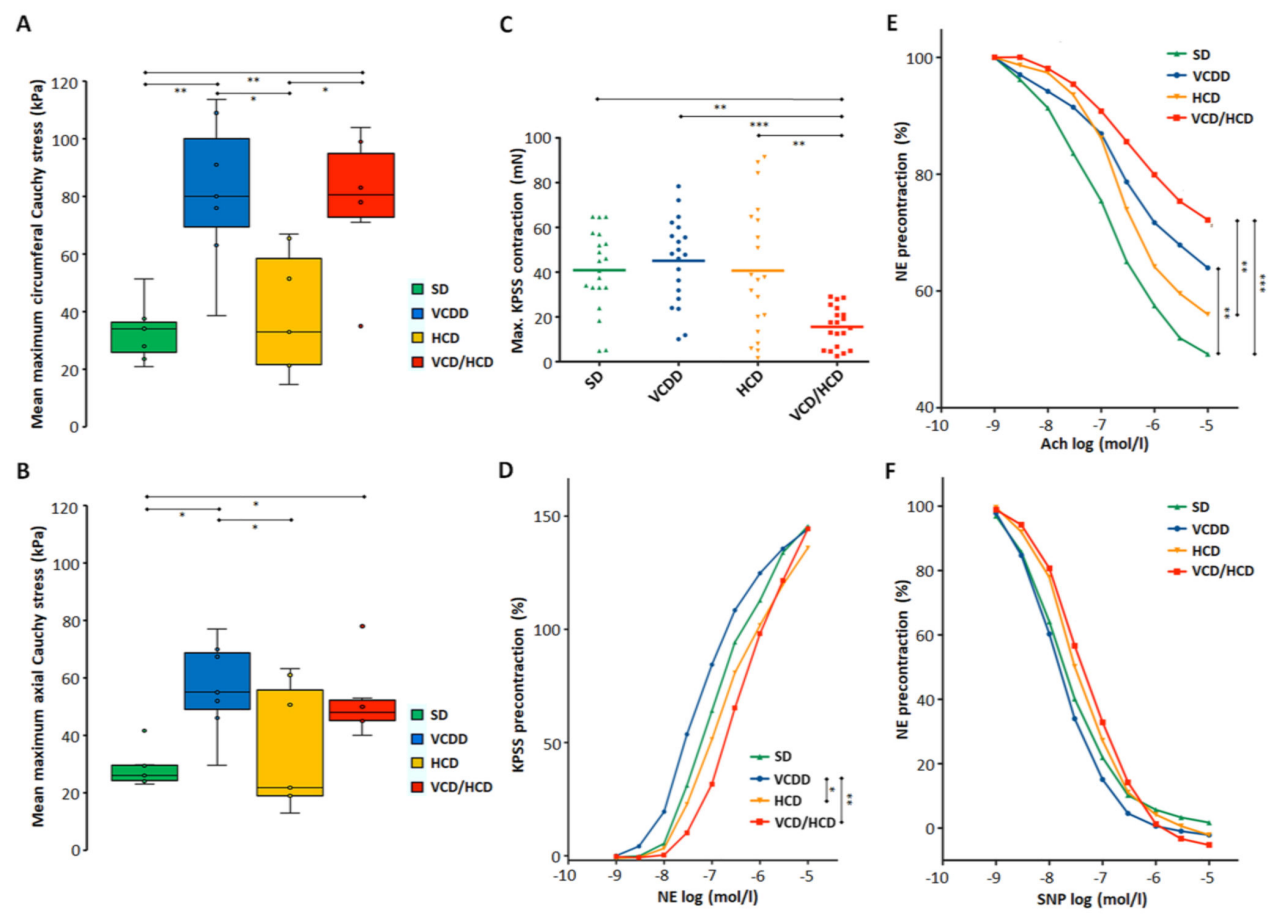
Biomechanical properties and vascular reactivity of aortic sections from rabbits fed different diets. A-B Biaxial mechanical properties in terms of Cauchy stress vs. stretch behavior in the circumferential (A) and longitudinal (B) directions, (n = 7). *p 0.05; **p 0.01; ***p 0.001. C–F Myographical analysis of freshly dissected aortas. Aortic sections of each rabbit were cut into 4 rings, contracted with modified Krebs-Ringer bicarbonate buffer solution containing high K^+^ concentration (KPSS) (C) and norepinephrine (NE) (D) and subsequently relaxed with acetylcholine (Ach) (E) and sodium nitroprusside (SNP) (F), repeated measurements. In total, 24 rings from 6 rabbits per group were used. In D-F mean values for each group are shown. *p 0.05; **p 0.01; ***p 0.001. Significant differences are shown in S7.

Biaxial extension tests revealed that VCDD induces mechanical stiffening of the aorta, even in the absence of hypercholesterolemia (Fig. 6A and B, Kruskal-Wallis-Test, SD-VCDD, H=13.780, p = 0.007, p_adj_=0.042 at 1.3 stretch circumferential direction and H=8.980, p = 0.035, p_adj_=0.208 at 1.4 stretch longitudinal direction, respectively and supplement 6). Under our study conditions, HCD did not lead to aortic stiffening (Fig. 6A and B, SD-HCD, p = 0.807, p_adj_=1.000 at 1.3 stretch circumferential direction and p = 0.944, p_adj_=1.000 at 1.4 stretch longitudinal direction; VCDD-VCD/HCD, p = 0.940, p_adj_=1.000 at 1.3 stretch circumferential direction and p = 0.806, p_adj_=1.000 at 1.4 stretch longitudinal direction and supplement 6). In contrast, VCDD resulted in significantly increased aortic stiffening in the circumferential direction when combined with HCD, compared to HCD alone (Fig. 6A and B, HCD-VCD/HCD, p = 0.011, p_adj_=0.066 at 1.3 stretch circumferential direction and supplement 6), suggesting that VCDD has a stronger impact on aortic elasticity than HCD. Furthermore, feeding rabbits VCD/HCD resulted in massive impairment of K^+^-induced contraction of the aortic rings compared to any single diet, indicating that the combination of VCDD and HCD is particularly harmful to the aorta (Fig. 6C, 1-way ANOVA, p 0.001, p_adj_ 0.001). While neither HCD nor VCD/HCD led to significant impairment of norepinephrine-reactivity of aortic rings compared to the chow diet (Fig. 6D, 2-way ANOVA, HCD-SD, p > 0.999, VCD/HCD-SD, p = 0.214; diet F(3,76)= 13.46, p 0.001), both HCD and VCD/HCD resulted in significantly impaired norepinephrine-reactivity compared to VCDD (Fig. 6D, HCD-VCDD, p = 0.017, VCD/HCD-VCDD, p = 0.002), suggesting a role of VCDD in norepinephrine-induced contraction. Furthermore, while HCD had no effect on acetylcholine-relaxation of aortic rings compared to the chow diet (Fig. 6E, HCD-SD, p = 0.106; diet F(3,76)= 3.465, p = 0.020), VCDD and VCD/HCD resulted in significant impairment of acetylcholine-relaxation (VCDD-SD, p = 0.004, VCD/HCD-SD, p 0.001) suggesting a role of VCDD in acetylcholine-relaxation. Neither HCD, VCDD nor VCD/HCD had a significant effect on sodium nitroprusside-relaxation of aortic rings compared to SD (Fig. 6F, SD-HCD, SD-VCDD, SD-VCD/HCD, p > 0.999; diet F(3,74)= 3.172, p = 0.029), further indicating that the damage was confined to the endothelium and did not affect the underlying smooth muscle cells. Wider spacing between elastic fibers and smooth muscle cells in the media in response to VCD/HCD (Fig. 5D) suggest a direct link to impaired vascular reactivity. Altogether, our data indicate that VCDD already in the absence of HCD induces atherogenic transformation of the aorta, and aggravates atherosclerosis in its presence.

## 4 Discussion

Although atherosclerotic cardiovascular disease was previously considered to be a problem of industrialized countries, it is now common worldwide [18]. Today only half of all atherosclerosis cases can be explained by established risk factors, with many young or middle-aged individuals displaying asymptomatic and subclinical atherosclerotic lesions [1,18]. Despite the identification of a number of risk factors that drive arterial wall alterations, such as dyslipidemia, endothelial dysfunction, inflammation, immune disease, malnutrition and lifestyle [18] understanding of atherosclerosis development is still insufficient. A deeper understanding of new risk factors for atherosclerosis as well as the development of novel concepts of atherogenesis that include these factors are urgently needed to improve treatment of cardiovascular disease, and to help in understanding its interplay with associated diseases such as COVID-19 [19].

Preclinical animal models play a central role in atherosclerosis research [2]. Due to similarities to human lipoprotein metabolism the rabbit is a good model for studying the interaction of various atherosclerotic risk factors [4]. HCD is a potent trigger of atherogenesis in preclinical animal models; however, HCD can be toxic over prolonged periods of time. Injury of the arterial wall accelerates formation of advanced lesions in response to hyperlipidemia, helping to overcome the toxic effects [4]. Nonetheless, the commonly used classical injury techniques lead to non-reproducible/uneven atherosclerotic plaque development, thereby limiting the potential of such models.

To standardize atherosclerotic rabbit models, we have developed an automated injury technique (JURY) that renders the formation of atherosclerotic lesions more reproducible and homogeneous. The JURY device compensates for anatomical differences in vessel diameter and keeps friction more constant due to automated retraction. JURY is connected to a computer enabling adjustment of injury parameters, recording and display of balloon pressure data on a monitor in real-time. This technique induces mild injury already at 1.2 bar and single retraction, limiting the injury to the endothelial layer and reducing undesirable effects such as aortic aneurysms, which can be caused by high pressure and/or repeated denudation steps. Importantly, the automation of the vessel wall injury leads to complete denudation of the endothelium and more homogeneous and reproducible atherosclerotic plaque formation compared to manual injury. Balloon pressure is indeed a critical parameter influencing not only the severity of vascular injury but also subsequent intimal hyperplasia and vasomotor responses [5]. The damage produced by our method corresponds to type II injury [20] and could be induced over the entire intended area, allowing a more comprehensive analysis and extended applicability of the model. Comparison of automated JURY-controlled injuries with the classical “blind” and manual pressure-adjusted denudation demonstrates that the automated injury outperforms the other techniques.

MRI analyses applied in this study were sensitive to detect significant differences between the various injury scenarios. Diffusion tensor imaging is frequently used to evaluate tissue integrity and can detect slight alterations earlier than conventional MRI imaging [21]. FA was already shown to be highly sensitive to changes in microstructural composition of the arterial tissue [17]. The high standard deviation observed in the thinner aortic walls of the SD group in our study could be due to difficulties in segmentation. Nevertheless, our data further suggest that FA is a sensitive indicator of arterial wall remodeling, offering new insights into reorganization of connective tissue during atherogenesis.

In the present study, the capabilities of the JURY device have been far from exhausted, and more sophisticated injury scenarios could be envisaged based on the results obtained. Indeed, repeated denudation and/or more severe injury parameters result in formation of complicated plaques and thrombi [22]; however, under the described injury conditions, thrombi were not observed in this study. The JURY device is superior in pressure maintenance and is, to our knowledge, the first motorized catheter retraction system. Furthermore, standardization of the injury method minimizes the number of experimental animals needed.

The incidence of atherosclerosis cannot be adequately explained by established risk factors [1]. Therefore, identifying and understanding further risk factors for atherosclerosis is of utmost importance. Mouse models widely used in preclinical atherosclerosis research, however, do not allow the potential proatherogenic effects of elevated Hcy to be assessed under conditions of unblocked but compromised lipoprotein remnant clearance characteristic for humans [8,9]. Not only Hcy itself, but also vitamins required for Hcy metabolism and associated with the occurrence of HHcy are still debated regarding their possible role in subclinical atherosclerosis and/or its progression [13,23]. Lipoprotein metabolism in rabbits is similar to that in humans [24,25]. Therefore, rabbits are a good model not only for studying hyperlipidemia-induced atherosclerosis or preclinical drug screening, but also for dissecting the role of Hcy, its associated metabolites as well as vitamins, choline or other risk factors for atherosclerosis development. Higher reproducibility of injury, more uniform and more extended area of denudation of the endothelial layer achieved with automated injury technique facilitates reducing the number of animals required for such studies.

We used a diet free from vitamin B_12_ and reduced in folate, vitamin B_6,_ and choline to block Hcy metabolization and to investigate the effects of this diet on the development of atherosclerosis in the absence and presence of hypercholesterolemia in rabbits. While our data do not allow to conclude whether depleted vitamins B_12_, B_6_ or folate, reduced availability of choline or accumulated Hcy is responsible for the changes observed, they clearly show that combining HCD and VCDD markedly increases atherogenic transformation of the aorta. Thus, these results suggest that hypercholesterolemia and interference with Hcy metabolism or other consequences of vitamin deficiency have synergistic effects on atherosclerosis development. In accordance, low serum vitamin B_12_ levels have previously been found to be associated with unfavorable lipid profiles in humans [26]. The massively impaired vascular reactivity in response to VCD/HCD further confirms that the combined diet is particularly harmful to the aorta. Moreover, our data link VCDD to exacerbated norepinephrine-induced contraction as well as impaired acetylcholine-relaxation, both probably a consequence of endothelial dysfunction and/or altered biomechanical properties of the aorta. Supporting this notion, Hcy was shown to be associated with endothelial dysfunction [27], and the combination of HCD and methionine, a precursor of Hcy, results in virtually abolished endothelium-dependent relaxation [28].

Furthermore, VCDD leads to atherogenic transformation of the aorta even in the absence of HCD. Feeding VCDD to balloon-injured rabbits results in aortic stiffening and accumulation of lipids and macrophages in the neointima, suggesting that VCDD can induce atherogenic transformation of the aorta independently of hypercholesterolemia. Aortic wall elasticity drops in atherosclerosis [29] and pulse wave velocity, which is increased in stiffer arteries, is an independent predictor of cardiovascular morbidity and mortality [29]. Compromised organization of collagen [25] or elastin [30], or vessel calcification [31], which are all linked to elevated Hcy, may induce aortic stiffening in response to VCDD. Noteworthy, elastin fragmentation in atherosclerotic mice leads to a highly unstable plaque phenotype [32] and reduced aortic strain [33].

Accumulation of lipids, macrophages and dilated ER, as well as aortic stiffening in the absence of hypercholesterolemia imply that VCDD induces both different and similar atherogenic pathways in the aortic wall compared to those induced by HCD. The lack of significant increase in plasma Hcy in response to VCDD may be because it is metabolized to S-adenosyl-L-homocysteine (AdoHcy) [34,35] and Hcy-thiolactone [25], which were not determined in this study. Nevertheless, our data clearly show that atherogenic transformation of the aorta occurs even under these mild conditions, either via disruption of Hcy metabolism and/or due to independent consequences of vitamin and choline deficiency. Importantly, this transformation occurs despite the absence of hypercholesterolemia suggesting an intrinsic role of VCDD in atherogenesis. In accordance, VCDD drastically aggravates atherosclerosis in the presence of hypercholesterolemia.

In accordance with accumulation of lipids in the neointima of rabbits fed VCDD, Hcy is linked to activation of sterol regulatory element-binding proteins as well as increased expression of genes responsible for cholesterol/triglyceride biosynthesis and uptake in vascular endothelial and aortic smooth muscle cells [36]. Since VCDD does not increase plasma triglyceride or total cholesterol levels, lipid accumulation in the aortic wall of rabbits fed VCDD suggests deregulation of the intracellular lipid metabolism. Further, supporting the deregulation of lipid metabolism, rabbits fed VCD/HCD exhibited an increase in both LDL- and VLDL-cholesterol as well as VLDL-triglyceride compared to HCD alone. In accordance, Hcy/AdoHcy has already been linked to elevated LDL-cholesterol [37], reduced apolipoprotein A-I expression [38], activation of adipocyte lipolysis leading to hepatic steatosis [39], increased VLDL secretion [36], as well as decreased PPARα/γ mRNA and protein levels [40]. Thus, VCDD may increase LDL-, VLDL-cholesterol, and VLDL-triglyceride levels by upregulating cholesterol/triglyceride biosynthesis in the liver and affecting lipoprotein synthesis and/or metabolism. Furthermore, elevated Hcy may activate matrix metalloproteinases (MMPs) by increasing reactive oxygen species, and may also trigger smooth muscle cell migration within the vessel via MMP-dependent collagen degradation [41]. Observation of macrophages in the neointima of rabbits fed VCDD in the absence of hypercholesterolemia is in accordance with macrophage infiltration and activation of inflammation. Identifying which factor/s (deficiency of vitamin B_12_, deprivation of vitamin B_6_, folate, or choline or an accumulation of Hcy) is/are responsible for the atherosclerotic transformation of the aorta in the absence of hypercholesterolemia, and for aggravating atherosclerosis in the presence of hypercholesterolemia, requires further studies.

In summary, significant thickening of the aortic wall, massive impairment of vascular reactivity, altered FA and increased accumulation of lipids and macrophages in the neointima indicate a synergistic impact of a HCD on atherogenic transformation when it is combined with VCDD. Deficiency of B vitamins required for Hcy degradation has also an independent impact on atherogenic transformation of the aortic wall, by leading to accumulation of lipids and macrophages in the aortic neointima, accumulation of dilated ER in smooth muscle cells and fibroblasts as well as loss of vessel elasticity. Thus, the described rabbit model of atherosclerosis clearly shows that there are other factors besides high cholesterol that can exacerbate atherosclerosis, as well as induce cholesterol-independent atherogenic changes in the aortic wall. The JURY technique in combination with this model offers new tools to advance atherosclerosis research and expand the horizon for new therapeutic strategies.

## Supplementary Material

Statistical analyses

Supplementary material

## Data Availability

Data will be made available on request.

## Abbreviations

SD: standard diet
HCD: high cholesterol diet
VCDD: vitamin and choline deficient diet
VCD/HCD: vitamin and choline deficient high cholesterol diet
Hcy: homocysteine
HHcy: hyperhomocysteinemia
LDL: low-density lipoprotein
VLDL: very low-density lipoprotein
MRI: magnetic resonance imaging
FA: fractional anisotropy
SHG: second harmonic generation
TPAF: two-photon excited autofluorescence
CARS: coherent anti-Stokes Raman scattering
