# Supplementary material for: Deficiency of B vitamins leads to cholesterol-independent atherogenic transformation of the aorta
Source: Biomed Pharmacother. Author manuscript; Available in PMC 2024 Dec 9. (PMC7617128; doi:10.1016/j.biopha.2022.113640)
Supplement: Supplementary material [file EMS193251-supplement-Supplementary_material.docx]

**Supplements**

**Supplement 1-6**

**Supplement 1: The JURY device**

The JURY device was designed to provide the most uniform vessel wall damage possible using a balloon catheter.


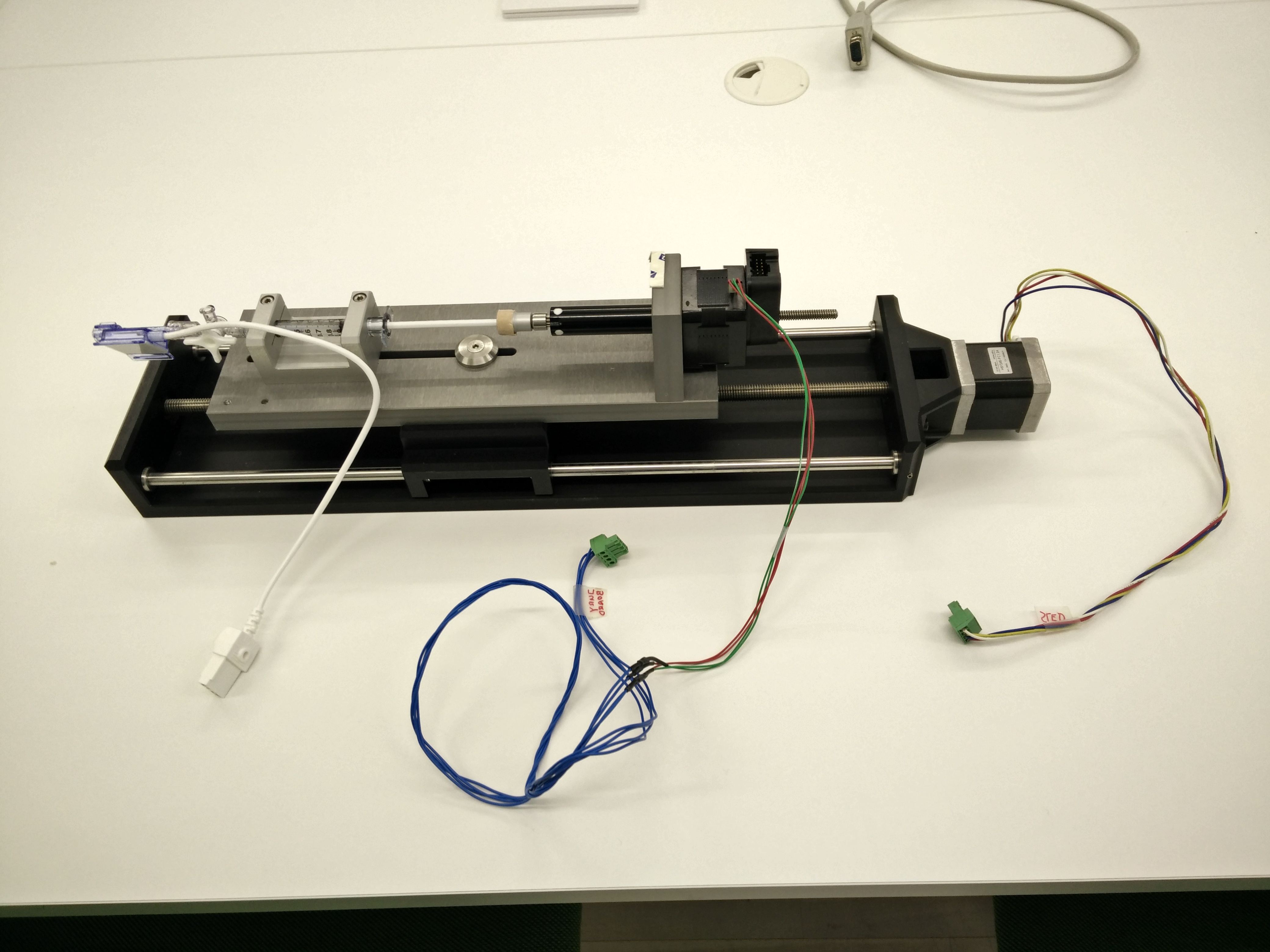


1

2

3

4

5

**Figure S1. The JURY device unplugged (latest version).** 1: pressure sensor; 2: 3-way stopcock/valve; 3: 1 ml Luer-Lock syringe; 4: linear actuator (catheter pressure regulation);

5: motor unit (catheter retraction).


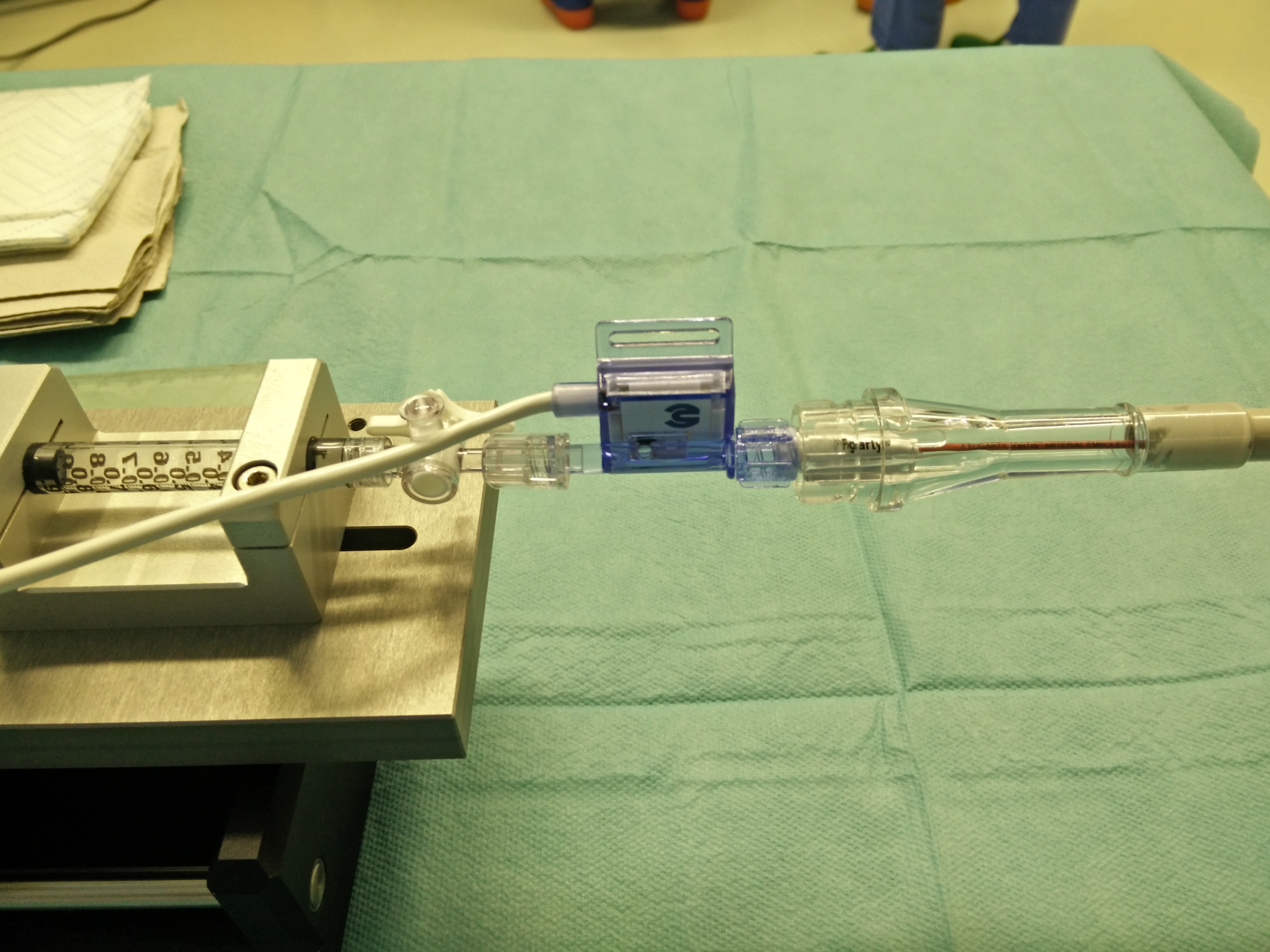


1

2

3

4

**Figure S2. The JURY device connected to the catheter.** 1: 1 ml Luer-Lock syringe;

2: pressure sensor; 3: 3-way stopcock/valve; 4: Fogarty catheter.


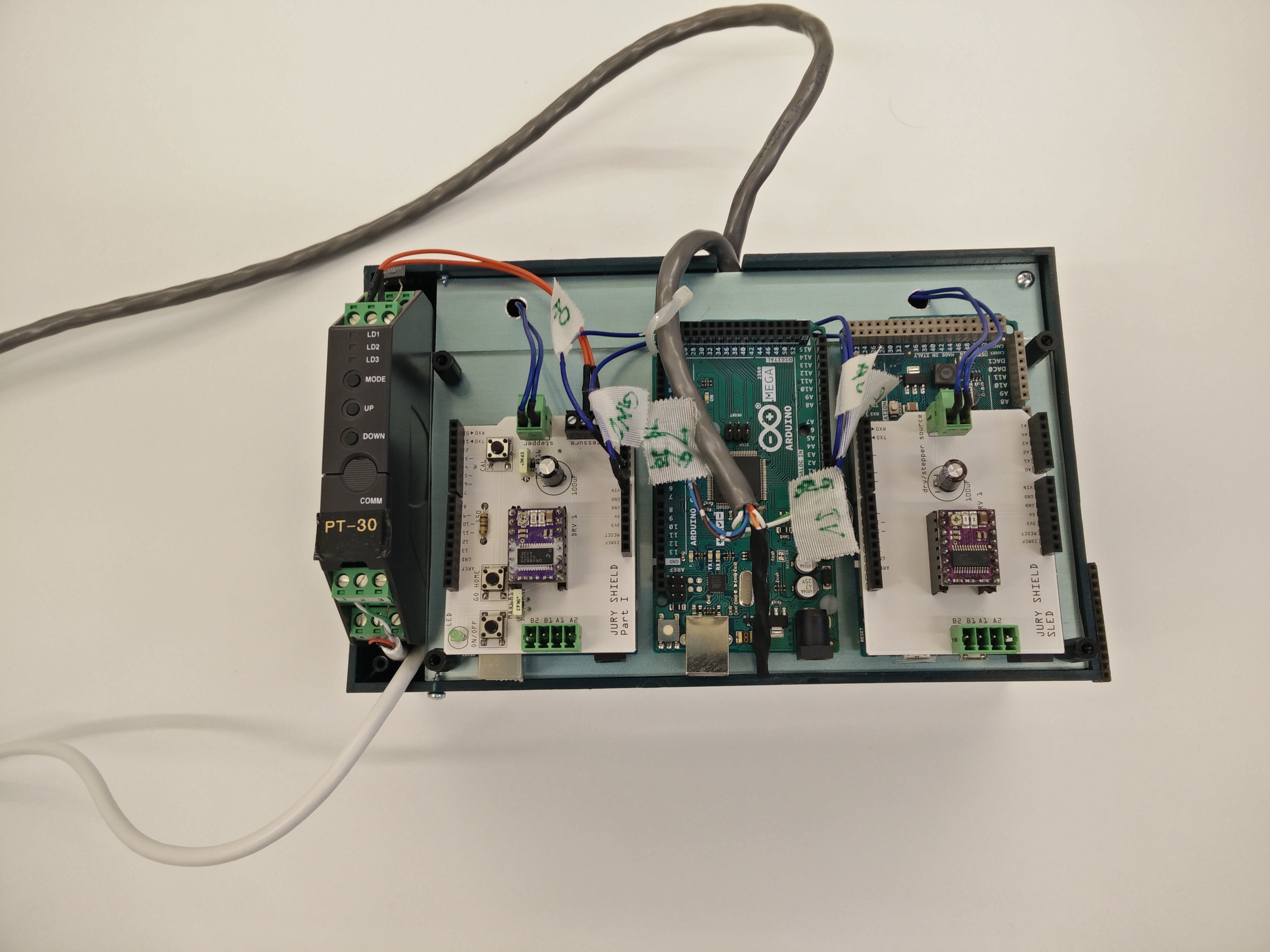


**Figure S3. JURY controller unit.**


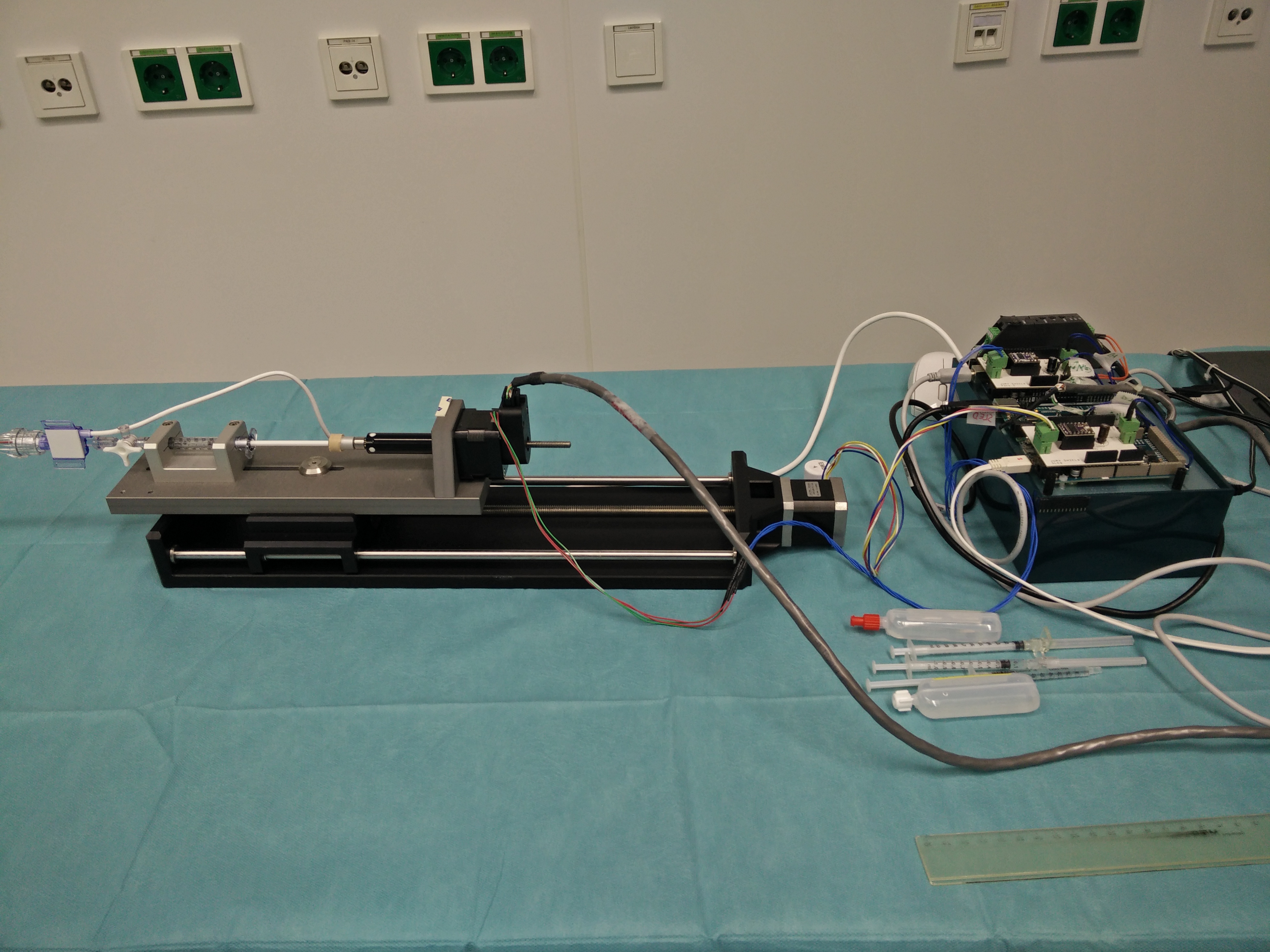


**Figure S4. JURY connected to the controller unit.**


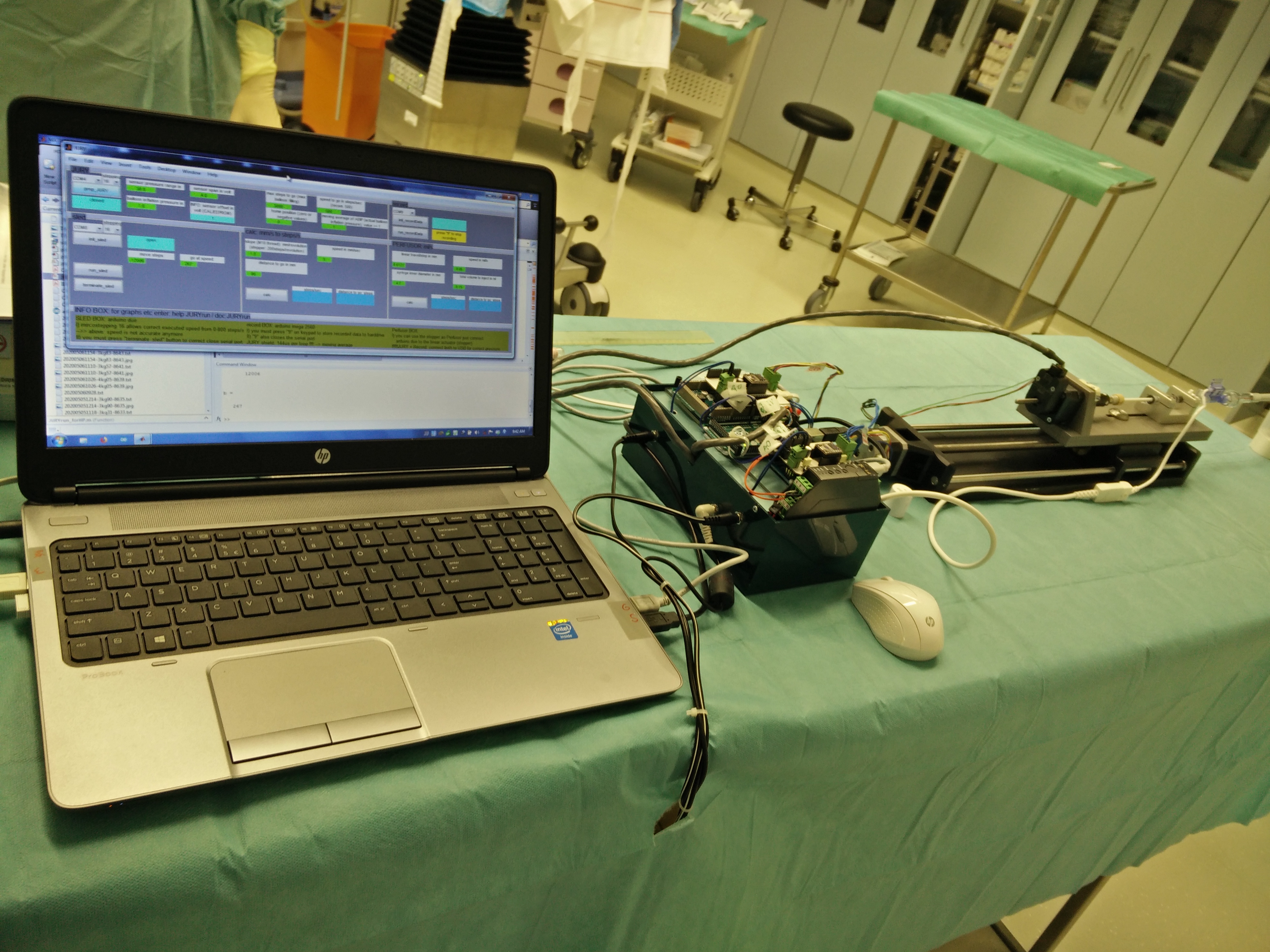


**Figure S5. JURY system ready for vessel wall injury.**


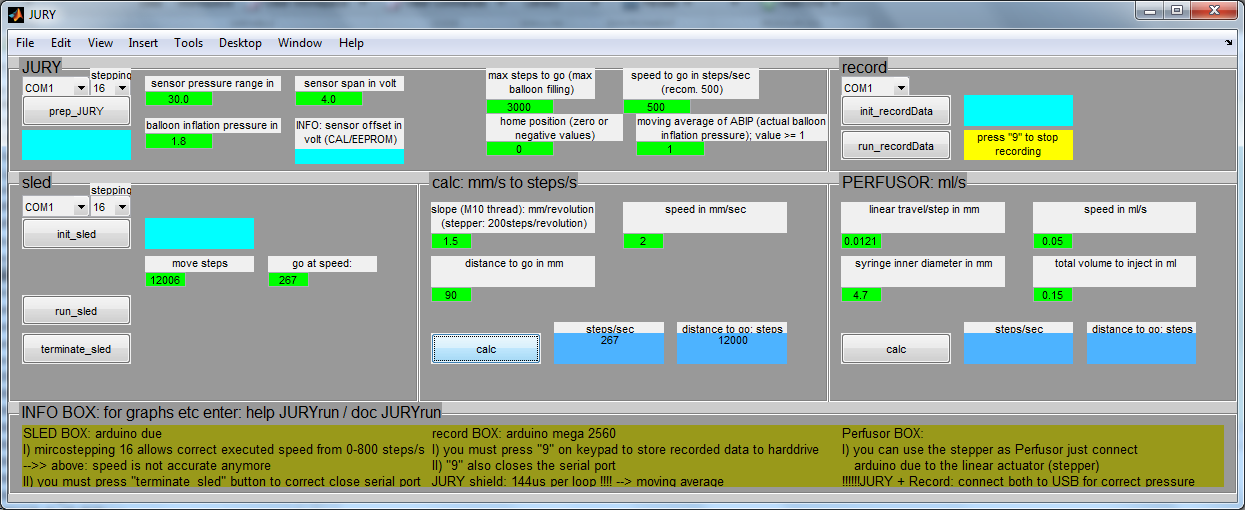


**Figure S6. JURY graphical user interface developed in MATLAB 2014.**

**Supplement 2: Vessel wall injury – surgical intervention**

Rabbits were anesthetized by intramuscular injection of Domitor® (Medetomidin), 0,25 mg/kg, and Ketasol® (Ketamin), 14 mg/kg. Then the rabbits were shaved at the area for incision (left groin) and placed on a heating pad to maintain body temperature. An eye gel was used to prevent dehydration of the cornea. Additionally, Rimadyl® (Carprofen), 4 mg/kg, and Baytril® (Enrofloxacin), 10 mg/kg, were injected subcutaneously for prevention of inflammation and infection. Next, the animals were intubated in abdominal position to control anesthesia and oxygen saturation during the surgical intervention. After fixation in dorsal position a vein catheter was introduced into the left ear to infuse fluid (5% Glucose, 20% Voluven, 20% Hydroxyethylstarch (HES), 55% Elomel with 10 ml/kg/hour). Before the incision the shaved area was disinfected and the surrounding covered by sterile drapes. The arteria femoralis was gently separated from all surrounding tissue with tweezers and exposed. Blood flow was interrupted from both sides by suture (Prolene 4.0).

**Proximal**


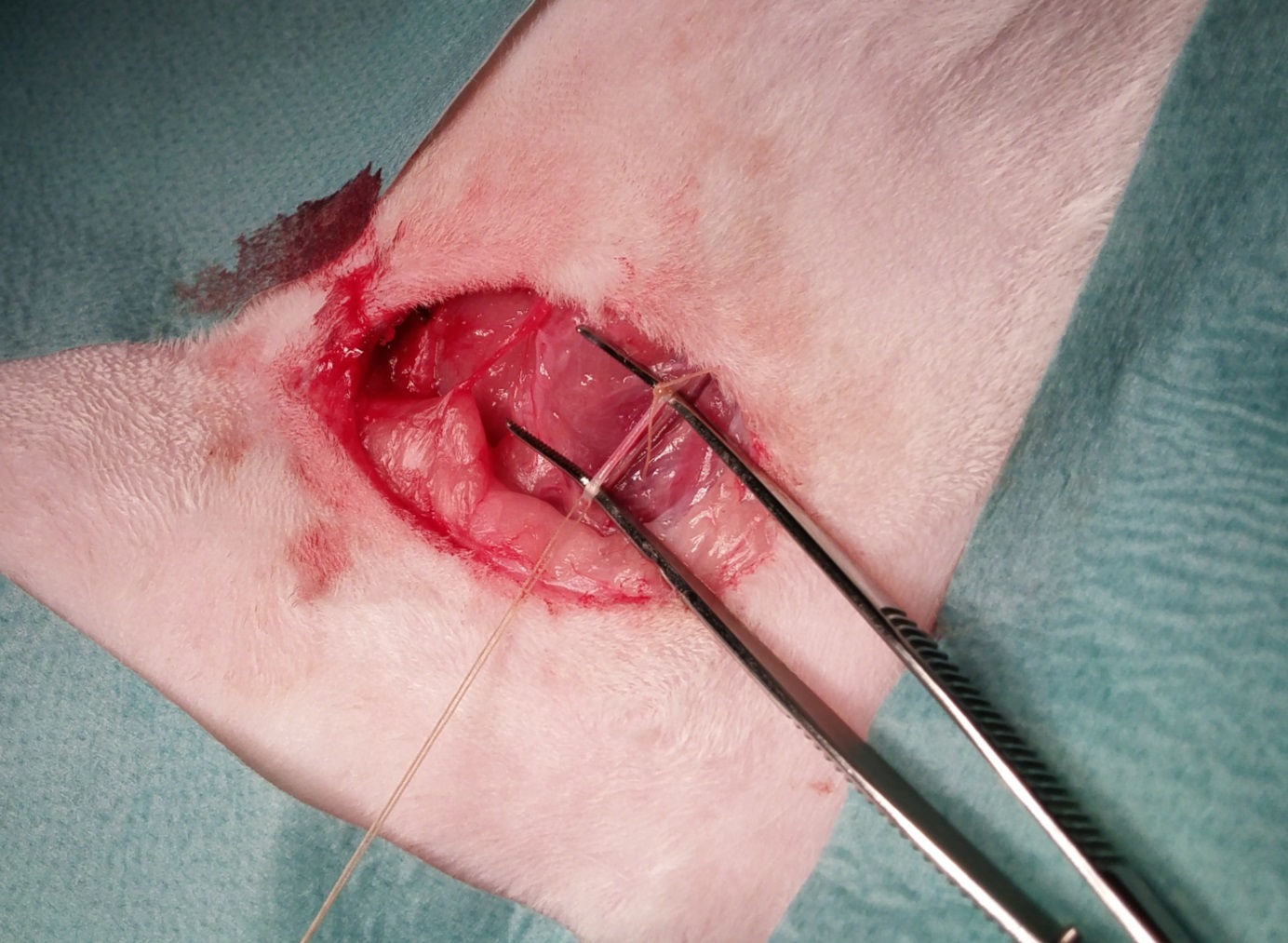


**1**

**1**

**Distal**

**Figure S7. Exposition of the arteria femoralis.** 1: medical suture.

Then the vessel was opened with micro scissors and a 5F gauge catheter sheath introducer was inserted and gently pushed up the arteria femoralis 7-8 cm into the arteria iliaca. Heparin, 200 I.E./kg, was administered over the ear vein catheter to prevent thrombosis.


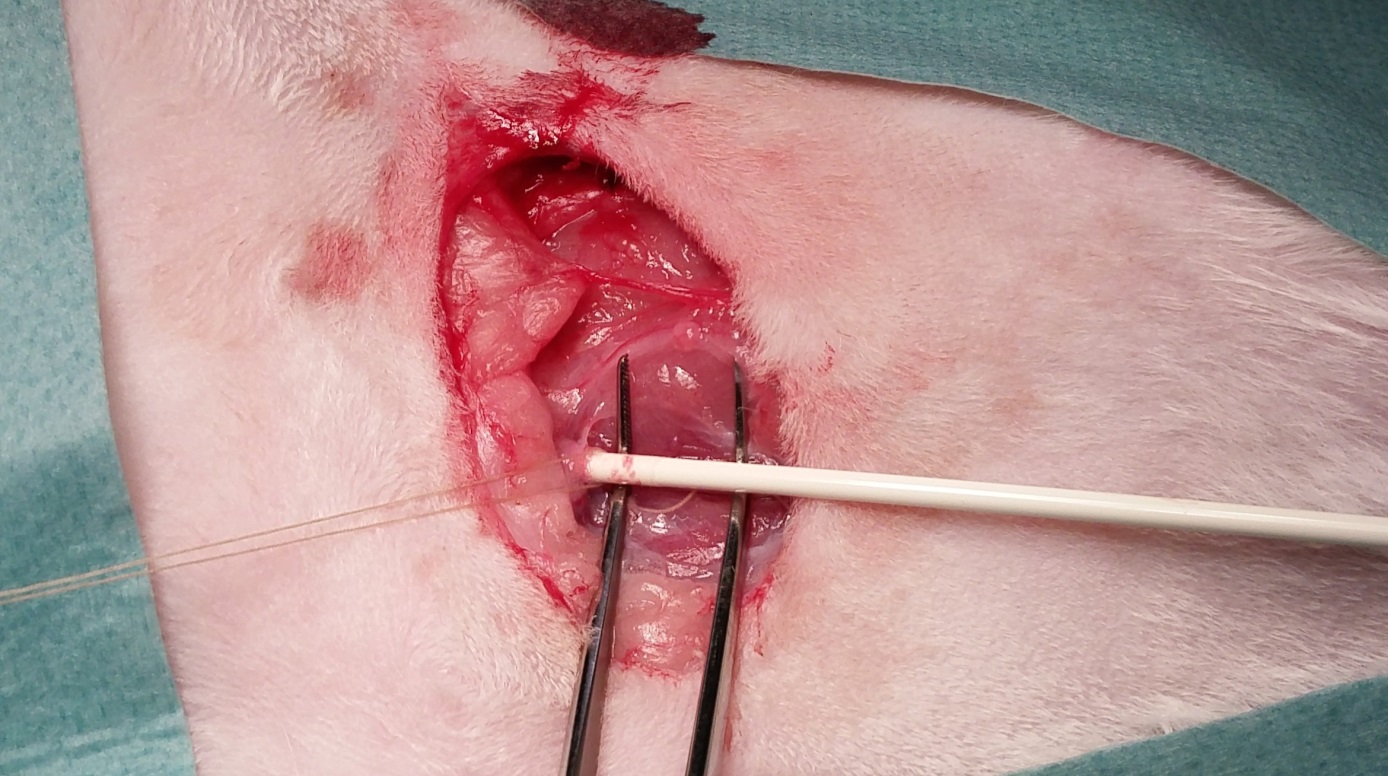


**Distal**

**Proximal**

**2**

**1**

**Figure S8. Introduction of the 5F gauge catheter sheath.** 1: medical suture; 2: 5F gauge catheter introducer sheath.

Next, the 4F catheter (filled bubble free with isotonic saline) was inserted into the introducer sheath and advanced 25 cm into the aorta abdominalis. Then the balloon denudation was performed either manually or controlled by the JURY device as shown in Figure S3. The exact site of injury was defined by imaging using a C-arm X-ray unit.


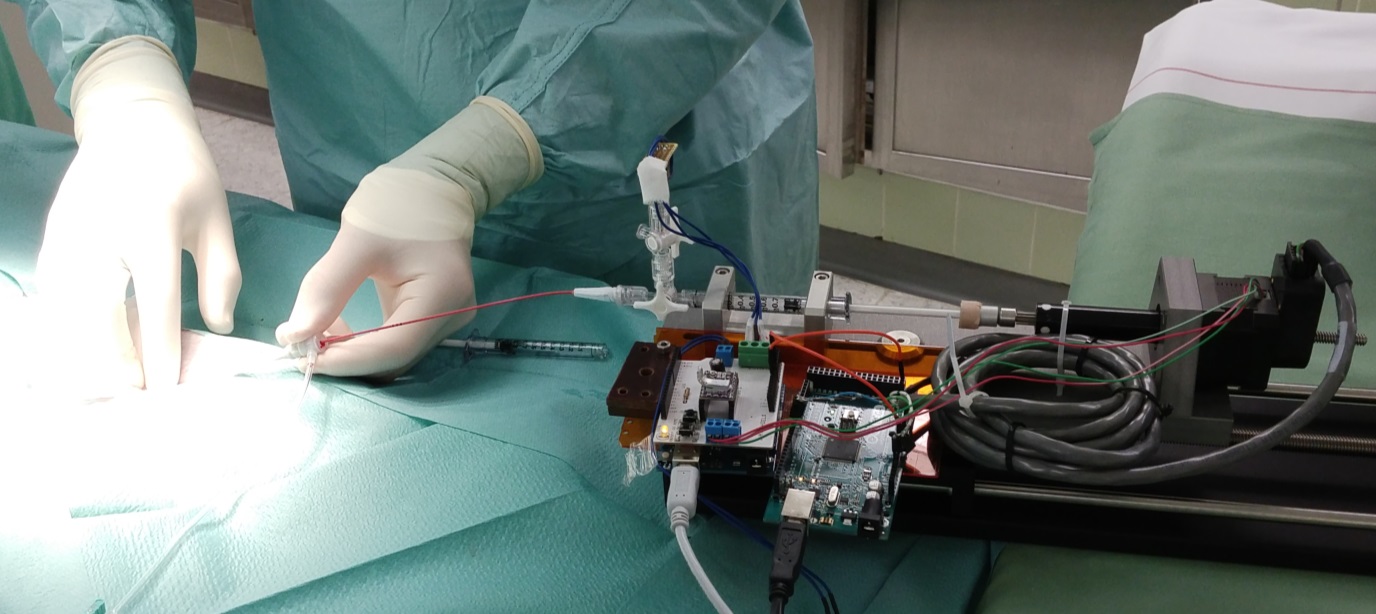


**4**

**2**

**3**

**1**

**Figure S9. Balloon denudation performed with the JURY device (previous version of the prototype).** 1: introducer sheath; 2: 4F balloon catheter; 3: 1 ml Luer-Lock syringe; 4: JURY device.

After the intervention both ends of the arteria femoralis were ligated with two sutures (Vicryl 4.0) proximal and distal the opening. Then the incision was sutured and treated adequately. For post operative analgetics Bupaq® (Buprenorphin), 0,05 mg/kg, was injected subcutaneously.

**Supplement 3**


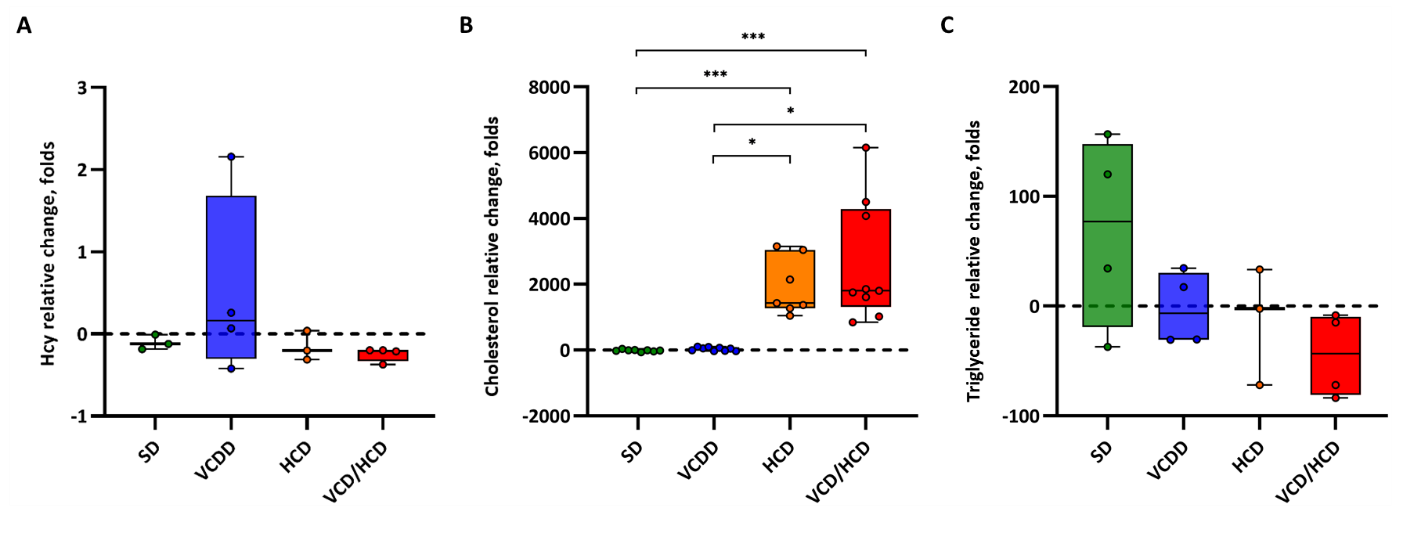


**Figure S10. Plasma Hcy, cholesterol and triglyceride levels in rabbits fed different diets.** Shown are relative changes of Hcy, n=3-4 (**A**), cholesterol, n=7-9 (**B**) and triglyceride, n=3-4 (**C**) plasma levels during the diets (before and after the diets). Points show single animals. SD – standard diet, VCDD - vitamin and choline deficient diet, HCD – high cholesterol diet, VCD/HCD – combined vitamin and choline deficient and high cholesterol diet.

**Supplement 4**


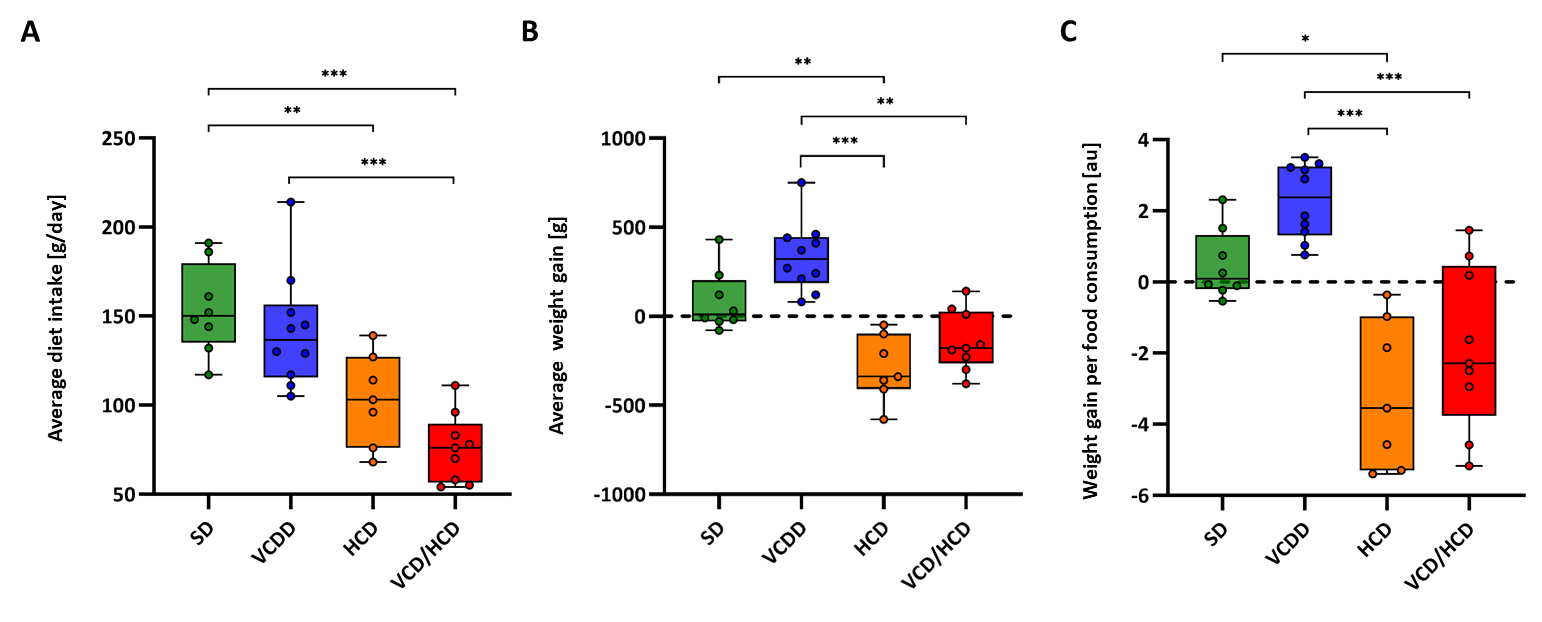


**Figure S11. Average diet intake and weight gain in rabbits fed different diets.**  au=arbitrary units. Points show single animals. SD – standard diet, VCDD - vitamin and choline deficient diet, HCD – high cholesterol diet, VCD/HCD – combined vitamin and choline deficient and high cholesterol diet, n=7-10**.**

**Supplement 5**


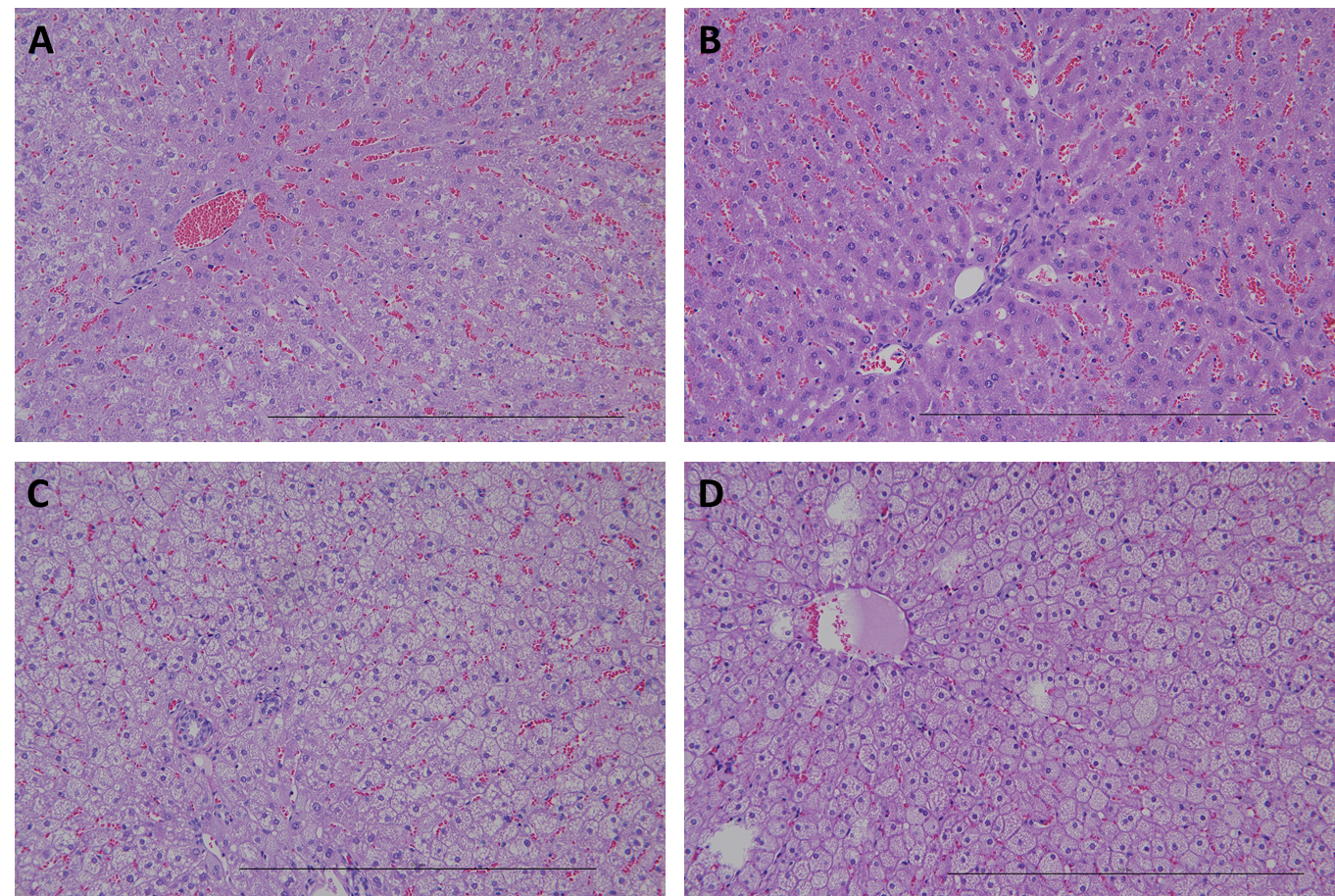


**Figure S12. Histological examination of liver specimens from rabbits fed different diets.** Representative images of HE stained liver sections. **A** SD – standard diet; **B** VCDD - vitamin and choline deficient diet; **C** HCD – high cholesterol diet, **D** VCD/HCD – combined vitamin and choline deficient and high cholesterol diet**.** HE=haematoxylin & eosin staining. Bars represent 500 µm.

**Supplement 6**


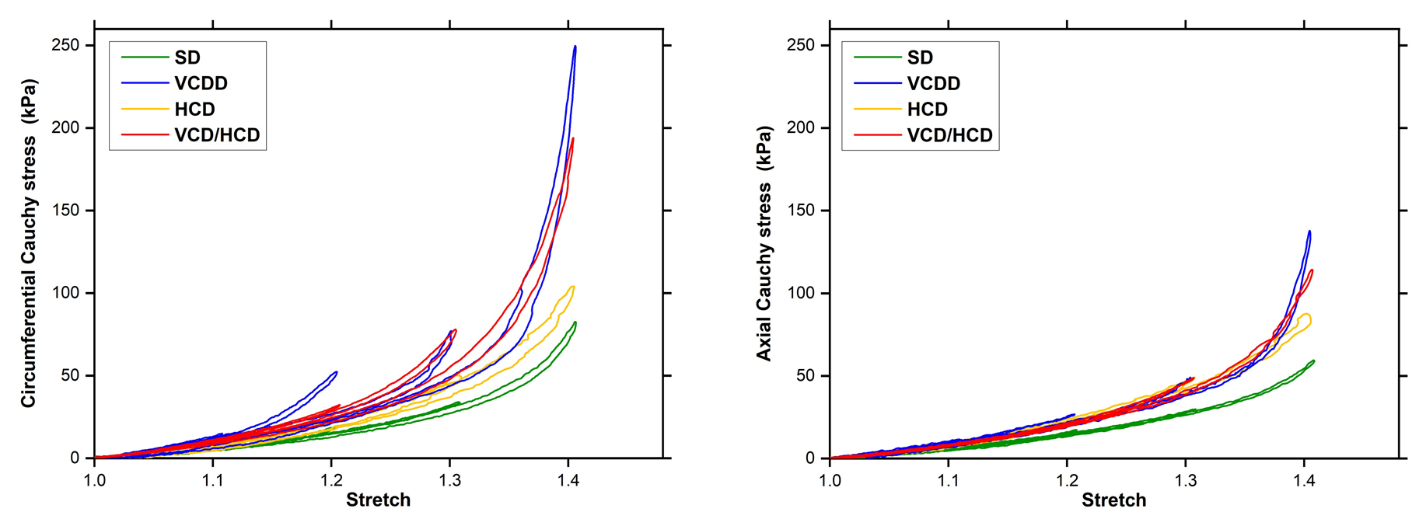


**Figure S13. Stretch curves from biomechanical testing of aorta sections from rabbits fed different diets.** Biaxial mechanical properties in terms of Cauchy stress vs. stretch behavior in the circumferential and longitudinal directions of representative aortas. SD – standard diet, VCDD - vitamin and choline deficient diet, HCD – high cholesterol diet, VCD/HCD – combined vitamin and choline deficient and high cholesterol diet.
